# A Review: Halogenated Compounds from Marine Fungi

**DOI:** 10.3390/molecules26020458

**Published:** 2021-01-16

**Authors:** Cong Wang, Huanyun Lu, Jianzhou Lan, KH Ahammad Zaman, Shugeng Cao

**Affiliations:** 1 Key Laboratory of Chemistry and Engineering of Forest Products, State Ethnic Affairs Commission, Guangxi Key Laboratory of Chemistry and Engineering of Forest Products, Guangxi Collaborative Innovation Center for Chemistry and Engineering of Forest Products, School of Chemistry and Chemical Engineering, Guangxi University for Nationalities, Nanning 530006, China; luhuanyun2020@163.com (H.L.); lanjianzhou1576@163.com (J.L.); 2 Department of Pharmaceutical Sciences, Daniel K. Inouye College of Pharmacy, University of Hawai’i, Hilo, HI 96720, USA; kzaman@hawaii.edu

**Keywords:** marine fungi, chemical structures, natural products, halogenated compounds

## Abstract

Marine fungi produce many halogenated metabolites with a variety of structures, from acyclic entities with a simple linear chain to multifaceted polycyclic molecules. Over the past few decades, their pharmaceutical and medical application have been explored and still the door is kept open due to the need of new drugs from relatively underexplored sources. Biological properties of halogenated compounds such as anticancer, antiviral, antibacterial, anti-inflammatory, antifungal, antifouling, and insecticidal activity have been investigated. This review describes the chemical structures and biological activities of 217 halogenated compounds derived mainly from *Penicillium* and *Aspergillus* marine fungal strains reported from 1994 to 2019.

## 1. Introduction

Marine fungi are a treasure source of marine natural products. Marine-derived fungi are important providers of biologically prominent natural products due to their ability to produce secondary metabolites with novel structures and pharmacological activities. According to a paper on marine microbial natural products from 2010 to 2013 [1], natural products from marine fungi account for 63% of marine microorganisms. Due to the enormous amount of chloride and bromide ions available in seawater, many of these secondary metabolites are halogenated. Marine natural products cover a diverse assembly of molecules, including polyketides, peptides, terpenes, phenols, acetogenins, alkaloids, and volatile halogenated hydrocarbons [2]. The fungi isolated from the marine sources might also be found in the terrestrial region. However, marine derived fungi usually produce more halogenated compounds than their terrestrial counterparts due to the presence of high halogen concentrations in the Ocean. Halogenated natural products encompass many classes of compounds, ranging in complexity from halocarbons (mostly halomethanes and haloethanes) to higher molecular weight molecules, which often contain oxygen and/or nitrogen atoms in addition to halogens [3,4]. One of the major focal points of research undoubtedly has been the discovery and characterization of new halogenated compounds, along with a remarkable effort toward the assessment of their possible pharmacological activities and biomedical applications. Active compounds account for nearly 59.2% new halogenated natural products isolated from marine fungi. This paper provides an overview of the sources of marine-derived fungi, chemical structures, and biological activities of 217 halogenated compounds (Table S1) derived from marine fungi from 1994 to 2019.

## 2. Halogenated Compounds from *Penicillium sp.*

### 2.1. Sponges-Associated Penicillium sp.

**Figure 1:**
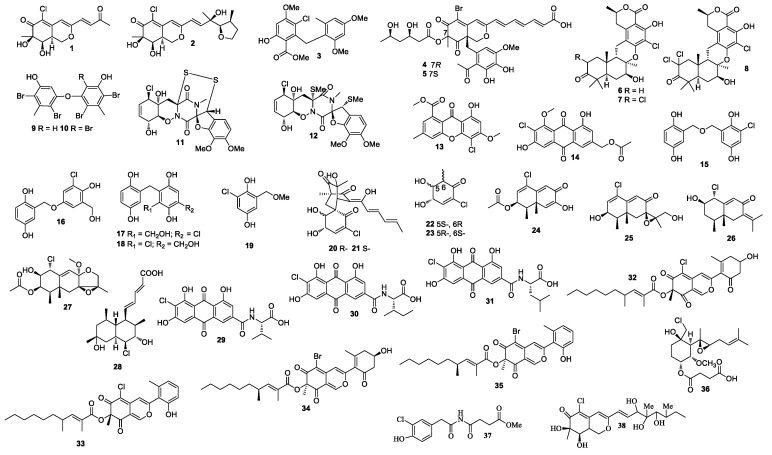
Structures of compounds **1–38**.

Two azaphilone derivatives penicilazaphilones D (**1**) and E (**2**) were isolated from a sponge-derived fungal strain *Penicillium sclertiorum* GDST-2013-0415 (Figure 1). Compound **2** was the first azaphilone with a tetrahydrofuran ring at C-3 [5]. A diphenyl ether methyl 3-chloro-2-(2,4-dimethoxy-6-methylphenoxy)-6-hydroxy-4-methoxybenzoate (**3**), bromophilones A (**4**) and B (**5**), were obtained from *Penicillium canescens* 4.14. 6a [6].

### 2.2. Other Marine Animals-Associated Penicillium sp.

New meroterpenoids chrodrimanins K and L (**6** and **7**) were separated from *Penicillium* sp. SCS-KFD09 (marine worm *Sipunculus nudus*), and **6** exhibited anti-H1N1 activity with an IC_50_ value of 74 μM [7]. A new meroterpenoid, named chrodrimanin O (**8**), was isolated from a fermentation of *Penicillium* sp. SCS-KFD09 (marine worm *Sipunculus nudus*). Compound **8** showed protein tyrosine phosphatase 1B inhibitory activity with an IC_50_ value of 71.6 μM [8].

### 2.3. Marine Algae-Associated Penicillium sp.

Diphenyl ethers 4,6,4′,6′-tetrabromo-3,3′-dihydroxy-5,5′-dimethyldiphenyl ether (**9**) and 4,6,2′,4′,6′-pentabromo-3,3′-dihydroxy-5,5′-dimethyldiphenyl ether (**10**) were obtained by feeding a culture of *Penicillium chrysogenum* with CaBr_2_. Compounds **9** and **10** showed 2,2-diphenyl-1-picrylhydrazyl (DPPH) activity with IC_50_ values of 18 and 15 μM, respectively [9].

### 2.4. Mangroves-Associated Penicillium sp.

Two new epipolythiodioxopiperazine alkaloids penicisulfuranols A (**11**) and D (**12**) with a rare spiro-furan ring, which were isolated from the mangrove endophytic fungus *Penicillium janthinellum* HDN13-309, showed cytotoxicity against Hela and HL-60 with IC_50_ values of 0.5 and 0.3, 0.1 and 1.2 μM, respectively [10]. In addition, 4-chloro-1-hydroxy-3-methoxy-6-methyl-8-methoxycarbonyl-xanthen-9-one (**13**) and 2′-acetoxy-7-chlorocitreorosein (**14**) were purified from the fungal strain *Penicillium citrinum* HL-5126, of which **14** showed activity against *Vibrio parahaemolyticus* with an MIC value of 10 μM [11].

### 2.5. Penicillium sp. from Marine Sediments

New gentisyl alcohol derivatives dimeric terrestrols B (**15**), D (**16**), F and G (**17** and **18**), and a monomer (**19**) were obtained from *Penicillium terrestre* and were cytotoxic toward HL-60, MOLT-4, A-549, and BEL-7402 with IC_50_ values in the range of 5.3 to 64.7 μM [12]. Compounds **15** and **16** exhibited tscavenging activity in a DPPH assay with IC_50_ values ranging from 4.1 to 5.2 μM. A study of the marine sediment derived fungus *Penicillium terrestre* resulted in the identification of chloctanspirones A (**20**), B (**21**), terrestrols K (**22**), and L (**23**). Compound **20** displayed inhibitory activity against HL-60 and A549 with IC_50_ values of 9.2 and 39.7 μM, respectively [13]. Compound **21** displayed inhibitory activity against HL-60 with an IC_50_ value of 37.8 μM. A chloro-trinoreremophilane sesquiterpene (**24**), and three chlorinated eremophilane-type sesquiterpenes (**25**–**27**) were purified from *Penicillium* sp. PR19N-1 isolated from the deep-sea sediment collected from Prydz Bay [14]. Compound **24** displayed inhibitory activity against HL-60 and A549 with IC_50_ values of 11.8 ± 0.2 and 12.2 ± 0.1 μM, respectively. Tanzawaic acid P (**28**) was isolated from a marine-derived fungal strain *Penicillium* sp. CF07370, and it was active against HeLa cell line with an IC_50_ value of 5.9 ± 0.8 µM after 72 h [15]. Emodacidamides C (**29**), F (**30**), and G (**31**) were obtained from a marine-derived fungal strain *Penicillium* sp. SCSIO sof101. Compound **29** inhibited interleukin-2 secretion with an IC_50_ value of 4.1 μM [16]. Penicilones C (**32**) and D (**33**) were purified from *Penicillium janthinellum* HK1-6, which were active against methicillin-resistant *S. aureus* (MRSA, ATCC 43300, ATCC 33591, ATCC 25923, ATCC 29213) and *E. faecalis* (ATCC 51299, ATCC 35667) with MIC values ranging from 3.13 to 12.5 μg/mL [17]. *Penicillium janthinellum* HK1-6 produced two azaphilones penicilones G (**34**) and H (**35**), which were active against MRSA (ATCC 43300, ATCC 33591, ATCC 25923, ATCC 29213) and *E. faecalis* (ATCC 51299, ATCC 35667) with MIC values in the range of 3.13–50 μg/mL [18].

### 2.6. Penicillium sp. from Other Marine Sources

Ligerin (**36**) was separated from *Penicillium canescentia* MMS351, which showed cytotoxicity against the POS1 cell with an IC_50_ value of 117 nM [19]. Ligerin was synthesized from fumagillin, and it showed good activity against SaOS2 [20]. The culture of *Penicillium copticola* TPU1270 (marine foam, Iriomote Island, Okinawa Prefecture, Japan) yielded penicillimide (**37**) [21]. A new azaphilone penicilazaphilone C (**38**), which was isolated from the fungus *Penicillium sclerotiorum* M-22, showed cytotoxicity against B-16 and SGC-7901 with IC_50_ values of 0.065 and 0.720 mM, respectively. Compound **38** also exhibited strong antibacterial activity against *Pseudomonas aeruginosa*, *Staphylococcus aureus*, *Escherichia coli,* and *Klebsiella pneumonia* with MIC values ranging from 0.037 to 0.150 mM [22].

## 3. Halogenated Compounds from *Aspergillus sp.*

### 3.1. Sponges-Associated Aspergillus sp.

**Figure 2:**
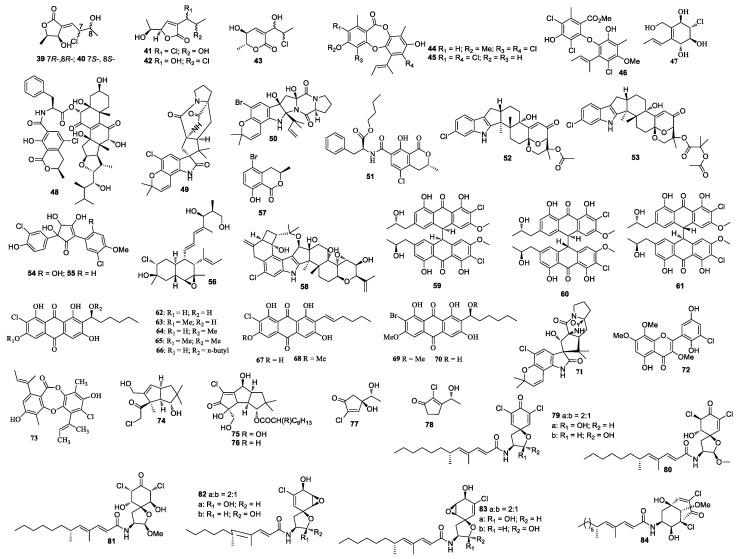
Structures of compounds **39–84**.

Two new polyketides chlorocarolides A (**39**) and B (**40**) were from *Aspergillus* cf. *ochraceus* 941,026 [23] (Figure 2). *Aspergillus ostianus* TUF 01F313 yielded 8-chloro-9-hydroxy-8,9-deoxyasperlactone (**41**), 9-chloro-8-hydroxy-8,9-deoxyasperlactone (**42**), and 9-chloro-8-hydroxy-8,9-deoxyaspyrone (**43**), of which compounds **42** and **43** inhibited the growth of *Ruegeria atlantica* at 25 μg/disc with an inhibition zone diameter of 10.1 and 10.5 mm, respectively [24]. Compound **41** was active against *Regenia atlantica* with an inhibition diameter of 12.7 mm at 5 μg/disc, and it was also active against *S. aureus* with an inhibition diameter of 10.2 mm at 25 μg/disc. Aspergillusidones B (**44**), C (**45**), and aspergillusether A (**46**) were separated from *Aspergillus unguis* CRI282-03 [25]. Compounds **44** and **45** inhibited aromatase with IC_50_ values of 4.1 and 0.7 μM, respectively. Compound **44** showed scavenging activity in a DPPH assay with an IC_50_ value less than 15.6 µM. *Aspergillus* sp. OUCMDZ-1583 (an unidentified marine sponge XD10410, Xisha Islands, China) produced a new metabolite, aspergone O (**47**), which inhibited α-glucosidase with an IC_50_ value of 1.54 mM [26]. Ochrasperfloroid (**48**) from *Aspergillus flocculosus* 16D-1 (the sponge *Phakellia fusca*, Yongxing Island, China) showed inhibitory activity towards THP-1 and NO production in LPS-activated RAW264.7, with IC_50_ values of 2.02 and 1.11 μM, respectively [27].

### 3.2. Other Marine Animals-Associated Aspergillus sp.

Notoamide N (**49**) and notoamide P (**50**) were isolated from the cultures of *Aspergillus* sp. MF297-2 [28,29]. A mycotoxin ochratoxin A n-butyl ester (**51**) was isolated from a marine-derived fungal strain *Aspergillus* sp. SCSGAF0093 from *Melitodes squamata* collected from the South China Sea. The bio-toxicity of compound **51** was determined by the brine shrimp lethality bioassay with a median lethal concentration (LC_50_) value of 4.14 μM [30]. Two new indole-diterpene alkaloids asperindoles A (**52**) and C (**53**) were isolated from the fermentation broth of *Aspergillus* sp. KMM 4676, of which compound **52** showed cytotoxicity toward PC-3 cells, LNCaP cells, and 22Rv1 cells, with IC_50_ values of 69.4, 47.8, and 4.86 µM, respectively [31].

### 3.3. Marine Algae-Associated Aspergillus sp.

*Aspergillus sydowii* produced sydowins A (**54**) and B (**55**) [32]. A polyoxygenated decalin derivative dehydroxychlorofusarielin B (**56**) was isolated from the culture of *Aspergillus* sp. MFB024, which exhibited antibacterial activities against *S. aureus*, MRSA, and *multidrug-resistant S. aureus* with an equal MIC of 62.5 μg/mL [33]. (*R*)-(–)-5-bromomellein (**57**), produced by *Aspergillus ochraceus*, exhibited radical scavenging activity against DPPH with an IC_50_ value of 24 μM [34]. *Aspergillus nidulans* EN-330 afforded a chlorinated indole-diterpenoid 19-hydroxypenitrem A (**58**), which inhibited cytotoxic activity against brine shrimp with a LD_50_ value of 3.2 μM and showed antibacterial activities [35]. *Aspergillus alliaceus* afforded allianthrones A–C (**59**–**61**), among which **59** displayed cytotoxic activity against the HCT-116 and SK-Mel-5 with IC_50_ values of 9.0 and 11.0 μM, respectively [36].

### 3.4. Aspergillus sp. from Marine Sediments

A study on the *Aspergillus* sp. SCSIO F063 derived from the marine sediment sample resulted in the discovery of chlorinated anthraquinones (1′*S*)-7-chloroaverantin (**62**), (1′*S*)-6-*O*-methyl-7-chloroaverantin (**63**), (1′*S*)-1′-*O*-methyl-7-chloroaverantin (**64**), (1′*S*)-6,1′-*O*,*O*-dimethyl-7-chloroaverantin (**65**), (1′*S*)-7-chloroaverantin-1′-butyl ether (**66**), 7-chloroaverythrin (**67**), 6-*O*-methyl-7-chloroaverythrin (**68**), brominated anthraquinones (1′*S*)-6,1′-*O*,*O*-dimethyl-7-bromoaverantin (**69**), and (1′*S*)-6-*O*-methyl-7-bromoaverantin (**70**) [37], of which compounds **63**, **64,** and **70** exhibited cytotoxic activities against SF-268 with MIC values of 7.11 ± 0.14, 34.06 ± 2.98, and 24.69 ± 0.72 µM, respectively. Compounds **63**, **64,** and **70** also showed cytotoxic activities against NCI-H460 with MIC values of 7.42 ± 0.14, 37.19 ± 1.95, and 18.91 ± 1.43 µM, respectively. Compounds **63**, **64,** and **70** further demonstrated cytotoxic activities against MCF-7 with MIC values of 6.64 ± 0.36 to 49.53 ± 0.72 µM, respectively. The deep-sea-derived fungal strain *A. westerdijkiae* DFFSCS013 afforded a new prenylated indole alkaloid 5-chlorosclerotiamide (**71**), which showed cytotoxicity against K562 with an MIC value of 44 μM [38]. 

### 3.5. Aspergillus sp. from Other Marine Sources

5’-Hydroxychlorflavonin (**72**) was purified from *Aspergillus* sp. AF119 [39]. A new depsidone 7-chlorofolipastatin (**73**) was isolated from *Aspergillus ungui* NKH-007 collected in the Suruga Bay, which inhibited SOAT1 and SOAT2 isozymes [40].

## 4. Halogenated Compounds from Other Marine Fungi

### 4.1. Other Sponges-Associated Fungi

**Figure 3:**
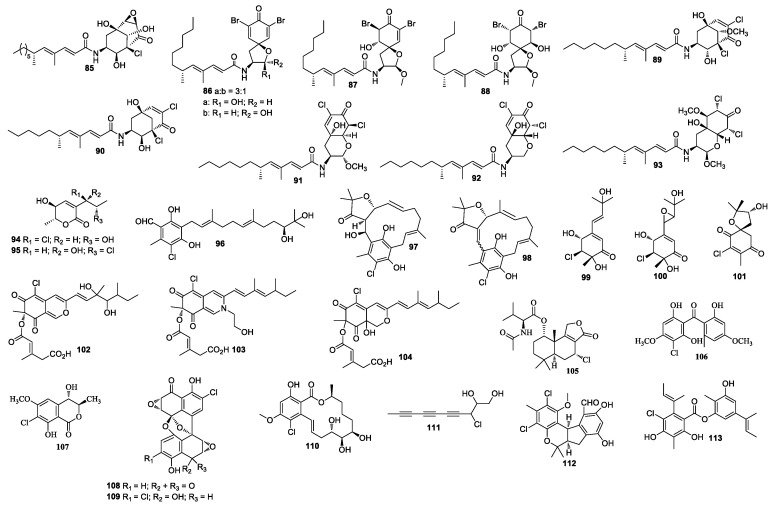
Structures of compounds **85–113**.

Cultivation of an unidentified fungal strain afforded three new chlorinated sesquiterpenes chloriolins A–C (**74**–**76**). Compound **74** inhibited human tumor cell lines T-47D and SNB-75 with IC_50_ values of 0.7 and 0.5 μM, respectively [41]. Trichodenone B (**77**), and trichodenone C (**78**) isolated from *Trichoderma harzianum* OUPS-N115 exhibited anticancer activity against P388 with ED_50_ values of 1.21 and 1.45 μg/mL, respectively [42]. Trichodenones B and C were synthesized by Usami et al. [43]. Gymnastatins A–G (**79**–**85**) [44,45,46], I–K (**86**–**88**) [47], Q (**89**) and R (**90**) [48], and dankastatins A–C (**91**–**93**) [48,49] were isolated from the cultures of *Gymnascella dankaliensis*. Gymnastatin A (**79**) was synthesized by anodic oxidation of the corresponding phenols [50]. Gymnastatins F (**84**) and Q (**85**) were synthesized by the tandem Michael and aldol reaction [51] (Figure 3). These compounds (**79**–**93**) showed cytotoxicity against P388, among which compounds **86** and **87** exhibited cytotoxicity against 39 human cancer cell lines with the average of log GI_50_ at −5.77 and −5.71, respectively. Compound **86** exhibited strong cytotoxic effect against HBC-5, NCI-H522, OVCAR-3, and MKN1, while compound **87** strongly inhibited SF-539, HCT-116, NCI-H522, OVCAR-3, and OVCAR-8. Compound **89** showed cytotoxicity against 39 human cancer cell lines with mean log GI_50_ values at −4.81, which also demonstrated cytotoxicity against BSY-1 and MKN7 with mean log GI_50_ values at −5.47 and −5.17, respectively. Compound 93 showed cytotoxicity against the P388 cell line with an ED_50_ value of 57 ng/mL. In a 2008 report, chlorohydroaspyrones A and B (**94** and **95**) obtained from *Exophiala* sp. showed antibacterial activity against *S. aureus* and multidrug-resistant *S. aureus* with an equal MIC value of 62.5 and 125 μg/mL [52]. Both compounds **94** and **95** demonstrated antimicrobial activity against MRSA with MIC values of 125 and 62.5 μg/mL, respectively. A culture of *Acremonium* sp. J05B-1-F-3 produced compounds **96**–**98** [53]. 5-Chloroacremines A and H (**99** and **100**), acremine O (**101**) were obtained from *Acremonium persicinum* [54]. New chloroazaphilone derivatives helicusin E (**102**), isochromophilone X (**103**), and isochromophilone XI (**104**) were isolated from *Bartalinia robillardoides* LF550. Compound **104** displayed antibacterial activity against *Bacillus subtilis, Staphylococcus lentus,* and *Trichophyton rubrum* with IC_50_ values of 55.6, 78.4, and 41.5 μM, respectively. Compounds **103** and **104** showed inhibitory activity against PDE4 with IC_50_ values of 11.7 and 8.30 μM [55], respectively. Minioluteumide A (**105**) was isolated from *Talaromyces minioluteus*, which showed weak cytotoxic activity [56]. Stachybogrisephenone B (**106**) was isolated from *Stachybotry* sp. HH1 ZSDS1F1-2, which displayed inhibitory activity against intestinal virus EV71 with an IC_50_ value of 30.1 μM and inhibited cyclooxygenase with an IC_50_ value of 8.9 μM [57]. One new isocoumarin derivative **107** was separated from *Phoma* sp. 135 [58], which was isolated from the sponge *Ectyplasia perox* collected in Dominica, Lauro Club Reef.

### 4.2. Other Marine Animals-Associated Fungi

**Figure 4:**
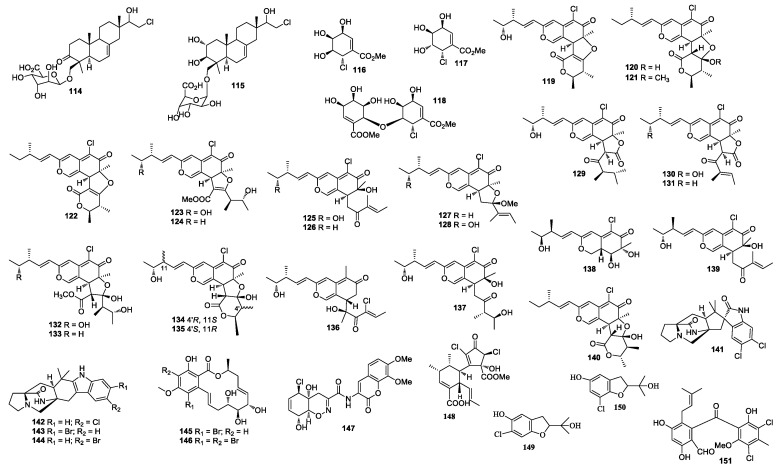
Structures of compounds **114–151**.

A marine-derived fungus LL-37H248 produced spiroxins A (**108**) and E (**109**). Compound **108** showed growth inhibition against 25 cancer cell lines with a mean IC_50_ value of 0.09 μg/mL [59]. The total synthesis of spiroxin A has been achieved in two competing cascade processes [60]. Cochliomycin C (**110**) was obtained from *Cochliobolus lunatus*, which was isolated from the gorgonian coral *D. gemmacea* [61]. Cochliomycin C (**110**) was synthesized from sugar D-lyxose [62]. Chondrosterin H (**111**) was purified from *Chondrostereum* sp. nov. SF002. The fungal strain SF002 was isolated from the coral *Sarcophyton tortuosum* [63]. A new chlorinated benzophenone derivative named (±)-pestalachloride D (**112**) was obtained from *Pestalotiopsis* sp. ZJ-2009-7-6 [64], which showed inhibitory activity against *Escherichia coli, Vibrio anguillarum,* and *V. parahaemoly-ticus* with MIC values of 5, 10, and 20 μM, respectively. (±)-Pestalachloride D was synthesized by way of a biomimetic Knoevenagel/Hetero-Diels–Alder Cascade reaction [65]. One new depside guisinol (**113**), which was active against *S. aureus* (5 mg/mL DMSO, 15 μL added), was identified from the metabolites of *Emericella unguis* M87-2 isolated from the cannonball jellyfish *Stomolopus meliagris* [66]. The chemical investigation of *Acremonium striatisporum* KMM 4401 from the sea cucumber *Eupentacta fraudatrix* [67] yielded two compounds, virescenosides Z_5_ and Z_7_ (**114** and **115**) (Figure 4). Pericosines A (**116**), D (**117**), and E (**118**) [68] were obtained from *Periconia byssaides* OUPS-N133, which was isolated from the sea hare *Aplysia kurodai*. Pericosine A (**116**) was synthesized from diverse aromatic *cis*-dihydrodiol precursors by the chemoenzymatic synthesis [69]. Compound **118** was synthesized by Mizuki et al. in 2014 [70]. *Chaetomium globosum* OUPS-T106B-6 isolated from the flathead grey mullet *Mugil cephalus* (Japan) yielded chaetomugilins C (**119**) [71,72], D-F (**120**–**122**) [73], G (**123**), H (**124**) [74], and I–O (**125**–**131**) [75], *seco*-chaetomugilins A (**132**) and D (**133**) [76], 11-*epi*-chaetomugilin A (**134**), 4’-*epi*-chaetomugilin A (**135**) [77], chaeto-mugilins P–R (**136**–**138**), 11-*epi*-chaetomugilin I (**139**) [78], chaetomugilin S (**140**), of which **119**–**122** were cytotoxic against P388 and HL-60 cell lines with IC_50_ values of 3.3–15.7 and 1.3–13.2 μM [71,72], respectively. Compounds **123**–**128**, **130**–**131,** and **134** showed growth inhibition against many cancer cell lines. (−)-Spiromalbramide (**141**), (+)-isomalbrancheamide B (**142**), (+)-malbrancheamide C (**143**), and isomalbrancheamide B (**144**) were produced by *Malbranchea graminicola* 086937A [79]. Two new brominated resorcylic acid lactones, 5-bromozeaenol (**145**) and 3,5-dibromozeaenol (**146**) [80] were produced by *Cochliobolus lunatus* TA26-46 induced by inhibitors of histone deacetylase. *C. lunatus* TA26–46 was isolated from the Zoanthid *Palythoa haddoni*. Trichodermamide B (**147**) was obtained from *Trichoderma virens* CNL910, which displayed cytotoxicity against HCT-116 with IC_50_ values of 0.32 μg/mL [81]. Compound **147** also showed inhibitory activity against *C. albicans*, *vancomycin-resistant E. faecium,* and MRSA with an equal MIC value of 15 μg/mL. The synthesis of **147** was reported by Lu and Zakarian in 2008 [82]. An unprecedented polyketide carbon skeleton roussoellatide (**148**) was obtained from the marine-derived fungus *Roussoella* sp. DLM33 [83]. Two benzofuran derivatives, 6-chloro-2-(2-hydroxypropan-2-yl)-2,3-dihydro-5-hydroxybenzofuran and 7-chloro-2-(2-hydroxypropan-2-yl)-2,3-dihydro-5-hydroxybenzofuran (**149** and **150**) were separated from *Pseudallescheria boydii*, which was isolated from the crown-of-thorns starfish *Acanthaster planci* (Hainan Sanya National Coral Reef Reserve, Hainan) [84].

### 4.3. Other marine Algae-Associated Fungi

**Figure 5:**
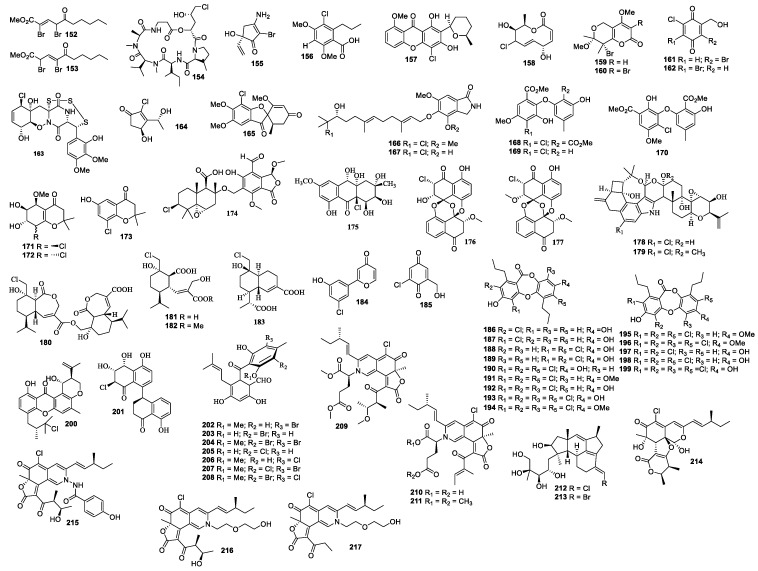
Structures of compounds **152–217**.

A new benzophenone pestalone (**151**) was isolated from a coculture broth of *Pestalotia* sp. CNL-365 and bacterium strain CNJ-328A. Compound **151** exhibited inhibitory activity against MRSA and vancomycin-resistant *Enterococcus faecium* with MIC values of 37 and 78 ng/mL, respectively. Compound **151** was cytotoxic against the NCI 60 human cancer cell lines with a mean GI_50_ value of 6.0 μM [85]. Compound **151** was synthesized with orcinol as the starting material [86]. Two new alkenoates, methyl 2,4-dibromo-5-oxo-2-decenoate (**152**) and methyl 2,4-dibromo-5-oxo-3-decenoate (**153**) were discovered from an unidentified fungus from the seaweed *Gracillaria verrucose* [87] (Figure 5). The chemical investigation of a culture of *Beauveria felina* yielded [β-MePro] destruxin E (**154**) [88]. A study of *Botrytis* sp. led to the identification of bromomyrothenone B (**155**) [89]. Acremonisol A (**156**) was obtained from *Acremonium* sp. [90]. Chaetoxanthone C (**157**) was separated from *Chaetomium* sp., which was active against *Trypanosoma cruzi* with an IC_50_ value of 1.5 μg/mL [91]. A 10-membered lactone (**158**) was isolated from a culture of *Curvularia* sp. 768 associated with the marine red algae *Acanthophora spicifera* [92]. Two new pyranopyranones, bromomethylchlamydosporols A (**159**) and B (**160**) were obtained from *Fusarium tricinctum*, which was active against SA, MRSA, and MDRSA with an equal MIC value of 15.6 μg/mL [93]. Bromochlorogentisylquinones A (**161**) and B (**162**) were isolated from *Phoma herbarum* and showed scavenging activity in a DPPH assay with IC_50_ values of 3.8 and 3.9 μM, respectively [94]. *Trichoderma* sp. (*cf. T. brevicompactum*) TPU199 in natural seawater medium supplemented with dimethyl sulfoxide afforded an unprecedented trithio-derivative of epidiketopiperazine, chlorotrithiobrevamide (**163**) [95]. One new trichodenone 3-hydroxytrichodenone C (**164**) was isolated from *Trichoderma asperellum* cf44-2 and showed antibacterial activities against four *Vibrio* strains with the inhibitory zone of 6.5–8.5 mm at 20 μg/disk. Compound **164** was active against *Prorocentrum donghaiense*, *Karlodinium veneficum*, *Heterosigma akashiwo,* and *Chattonella marina* with IC_50_ values of 37, 39, 35, and 30 μg/mL, respectively [96].

### 4.4. Other Mangroves-Associated Fungi

A new griseofulvin derivative 7-chloro-2’,5,6-trimethoxy-6’-methylspiro(benzofuran-2(3H),1’-(2) cyclohexene)3,4’-dione (**165**) was produced by *Sporothrix* sp. 4335 [97]. Emeriphenolicins A (**166**) and B (**167**) were produced by *Emericella* sp. HK-ZJ and were found to show antiviral activity with IC_50_ values of 42.1 and 62.0 μg/mL, respectively [98]. Pestalotethers A-C (**168**–**170**) and pestalochromones A-C (**171**–**173**) were purified from *Pestalotiopsis* sp. PSU-MA69, which was isolated from a branch of a mangrove plant *Rhizophora apiculata* [99]. Pestalotiopene C (**174**), a polyketide derivative, was obtained from *Acremonium strictum*, collected from the mangrove tree *Rhizophora apiculate* Blume [100]. *Paradictyoarthrinium diffractum* BCC 8704 produced a new hydroanthraquinone, paradictyoarthrin A (**175**), which showed cytotoxicity against KB, MCF-7, NCI-H187, and Vero with IC_50_ values of 26, 24, 23, and 31 μg/mL, respectively [101]. The marine mangrove *A. ilicifolius* provided *Lasiodiplodia theobromae* ZJ-HQ1, which produced chloropreussomerins A (**176**) and B (**177**). Compounds **176** and **177** showed antimicrobial activity against *S. aureus* and *B. subtili* with MIC values of 6.2, 50, 3.2, and 25 μg/mL, respectively. Compounds **176** and **177** also showed cytotoxicity against A549, HepG2, HeLa, MCF-7, and HEK293T with IC_50_ values ranging from 5.9 to 27 μM [102]. Rhizovarins A and B (**178** and **179**) were separated from a fermentation of *Mucor irregularis* QEN-189 isolated from *Rhizophora stylosa* (Hainan Island) and were cytotoxic against A-549 with IC_50_ values of 11.5 and 9.6 μM, respectively. Both compouds **178** and **179** were also cytotoxic to HL-60 cells with IC_50_ values of 6.3 and 5.0 μM, respectively [103]. Sesquiterpenoid derivatives, rhinomilisins A–C (**180**–**182**) and I (**183**) were isolated from *Rhinocladiella similis*, of which **180** showed cytotoxicity against L5178Y with an IC_50_ value of 5.0 μM [104].

### 4.5. Other Marine Plants-Associated Fungi

Polyporapyranone D (**184**) with a 2-phenylpyranon-4-one derivative skeleton was isolated from an extract of *Polyporales* sp. PSU-ES44 [105].

### 4.6. Other Marine Sediments-Associated Fungi

Chlorogentisylquinone (**185**) was purified from a marine-derived fungus FOM-8108, which showed nSMase activity with an IC_50_ value of 1.2 μM [106]. Spiromastixones B-O (**186**–**199**) were isolated from *Spiromastix* sp. MCCC3A00308, which exhibited antibacterial activity against *Staphylococcus aureus* ATCC 29213, *Bacillus thuringiensis* SCSIO BT01, and *Bacillus subtilis* SCSIO BT01 with MIC values in the range of 0.125–8.0 μg/mL. Compounds **190**–**194** exhibited activity against MRSA and *S. epidermidis* (MRSE) with the same inhibitory activity as levofloxacin. Compound **194** displayed inhibitory activity against VREF and VRE with an equal IC_50_ value of 4 μM [107]. Emerixanthone A (**200**) was isolated from *Emericella* sp. SCSIO 05240, which exhibited weak antibacterial activity against *Klebsiella pneumonia* (ATCC 13883), *Escherichia coli* (ATCC 29922), *Staphylococcus aureus* (ATCC 29213), *Aeromonas hydrophila* (ATCC 7966), *Acineto bacterbaumannii* (ATCC 19606), and *Enterococcus faecalis* (ATCC 29212) [108]. Cladosporol G (**201**) was purified from a fermentation of *Cladosporium cladosporioides* HDN14-342, which was isolated from a sediment sample (Indian Ocean). Compound **201** was cytotoxic against HeLa cell line with an IC_50_ value of 3.9 μM [109]. *Pestalotiopsis neglecta* yielded pestalones B–H (**202**–**208**), which were cytotoxic against PANC-1, A549, HCT116, MCFM, DU145, and HepG2 tumor cell lines with IC_50_ values in the range of 4.8–37 μM [110]. *Chaetomium globosum* HDN151398 yielded azaphilone alkaloids N-glutarylchaetoviridins A–C (**209–211**). Compound **211** exhibited cytotoxicity against HO8910 and MGC-803 with IC_50_ values of 6.6 and 9.7 µM, respectively [111].

### 4.7. Other Marine Source-Associated Fungi

A culture of *F. heterosporum* CNC-477 produced neomangicols A and B (**212** and **213**). Compound **212** was cytotoxic against MCF-7 and CACO-2 cells with IC_50_ values of 4.9 and 5.7 μM, respectively, and compound **213** showed antibacterial activity against *B. subtilus* at 50 μg/disc with an inhibition zone diameter of 10 mm [112]. Chaephilone C (**214**) and chaetoviridides A–C (**215**–**217**) were isolated from *Chaetomium* sp. NA-S01-R1. These compounds (**214**–**217**) showed antimicrobial activity and cytotoxicity [113].

## 5. Conclusions

**Figure 6:**
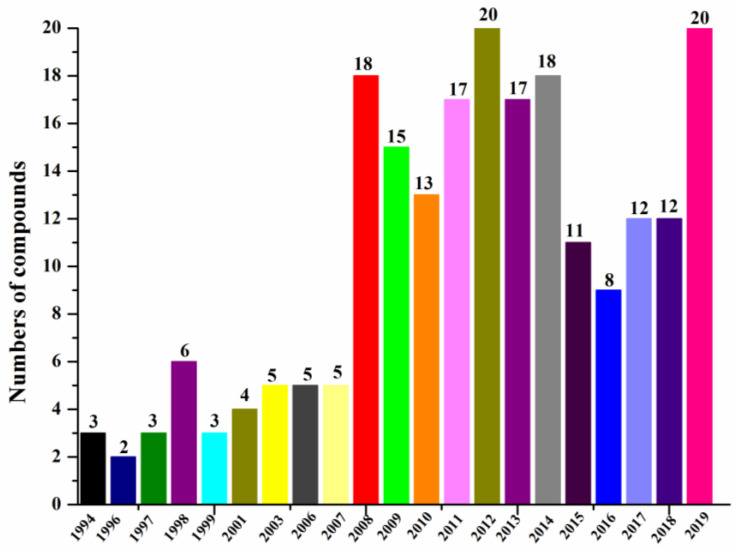
Numbers of new halogenated compounds reported annually from 1994–2019.

According to our summary of halogenated compounds identified from 1994 to 2019 (Figure 6, Table 1), the research on halogenated compounds from marine fungi was traced back to 1994 when chloriolins A–C (**74**–**76**) were discovered from an unidentified fungus isolated from the Indo-Pacific sponge *Jaspis aff. johnstoni* (Table 2) [41]. Since 2008, more new halogenated compounds than ever from marine fungi were isolated annually except before 2016. By the end of 2019, 217 new halogenated compounds from marine fungi have been reported. We have done our best to include as many new halogenated compounds isolated from marine fungi as possible, but the list may still not be complete.

**Figure 7:**
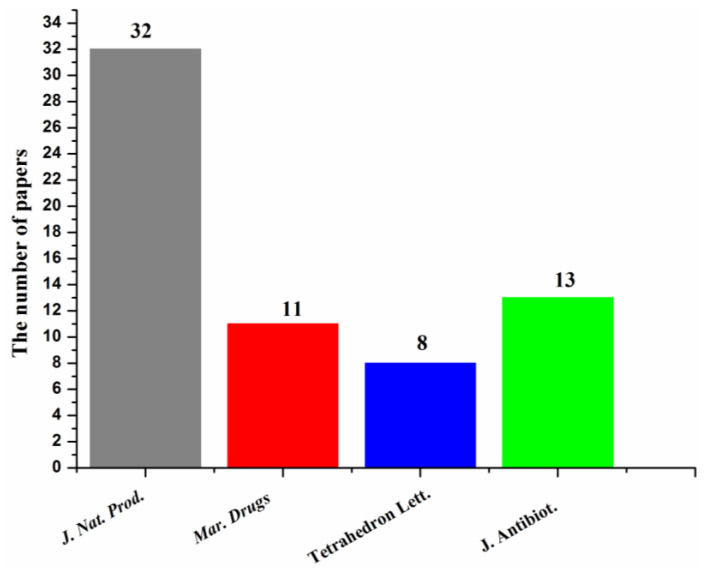
Journals that reported new halogenated compounds and numbers of papers published (1994–2019).

**Figure 8:**
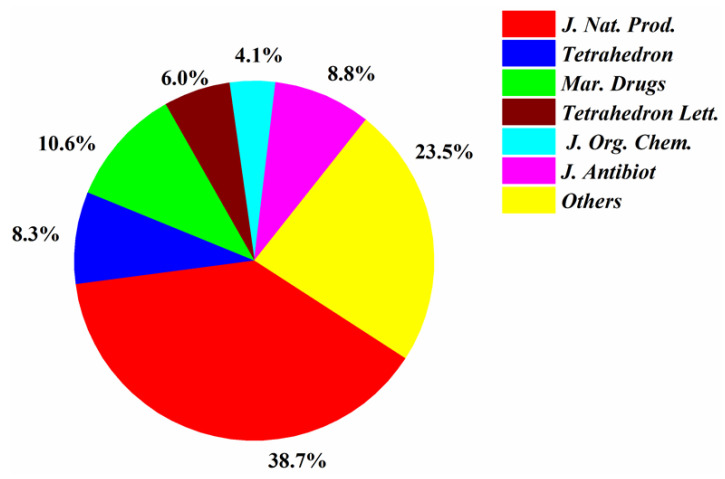
Percentages of new halogenated compounds published in different journals (1994–2019).

Most of the papers that reported new halogenated compounds in this period of time (1994–2019) were published in *J. Nat. Prod.* (32), *J. Antibiot*. (13), Marine Drugs (11), and *Tetrahedron Letters* (8) (Figure 7). The main journals that reported new halogenated compounds from marine fungi were *J. Nat. Prod.* (38.7%), *J. Antibiot*. (8.8%), *Tetrahedron* (8.3%), *Mar. Drugs* (10.6%), *Tetrahedron Lett.* (6.0%), and *J. Org. Chem.* (4.1%) (Figure 8). *J. Nat. Prod.* is the most preeminent journal that published more articles and more new halogenated compounds than any other journal.

**Figure 9:**
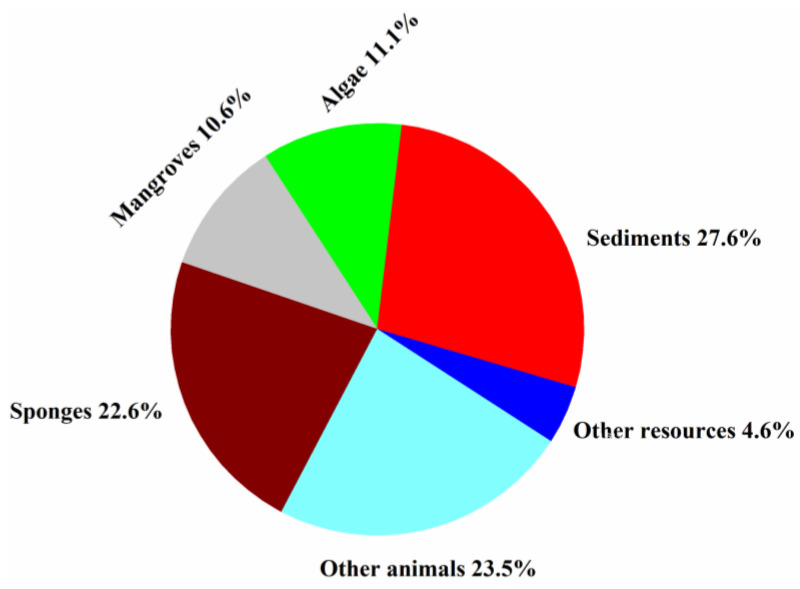
Percentages of new halogenated compounds from different sources of marine origins (1994–2019).

Fungi isolated from sponges, sediments, algae, and mangroves produced most of the new halogenated compounds (22.6, 27.6, 11.1, and 10.6%, respectively) (Figure 9). Marine animals hosted diverse fungal species and strains that produced more than 50% of the new halogenated compounds from 1994 to 2019, indicating that they are an excellent source for the discovery of new halogenated compounds.

**Figure 10:**
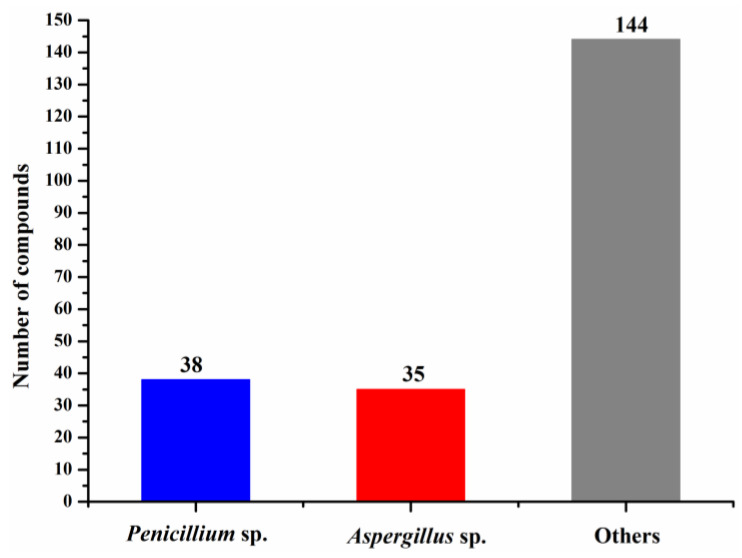
Numbers of new halogenated compounds from different marine fungi (1994–2019).

**Figure 11:**
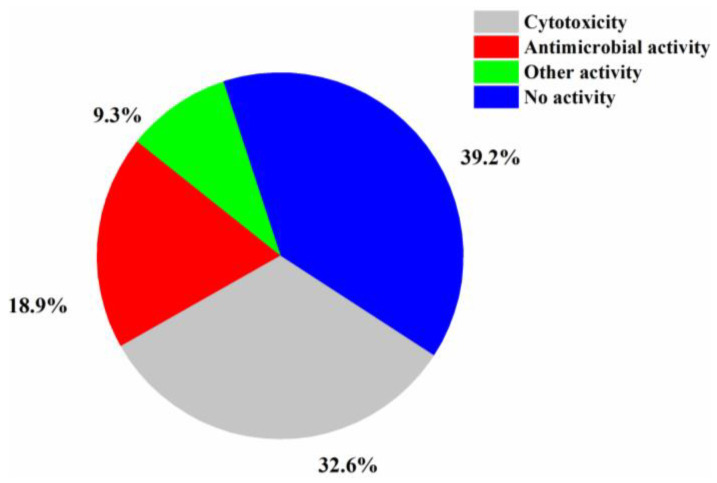
Activity of new halogenated compounds from marine fungi (1994–2019).

The numbers of halogenated compounds from marine *Penicillium* sp., *Aspergillus* sp., and the other fungi were 38, 35, and 144, respectively (Figure 10). It seems that halogenation in the marine environment is not specifically favorable to any fungal species or strains. Therefore, it would be interesting to investigate whether halogenations in marine fungi are enzymatic or nonenzymatic. The numbers of cytotoxic and antimicrobial halogenated compounds from marine fungi account for 32.6 and 18.9%, respectively (Figure 11). In addition, 39.2% of the halogenated compounds were tested as inactive in the reported assays, but it is worthy to evaluate these compounds in other biological settings.

**Figure 12:**
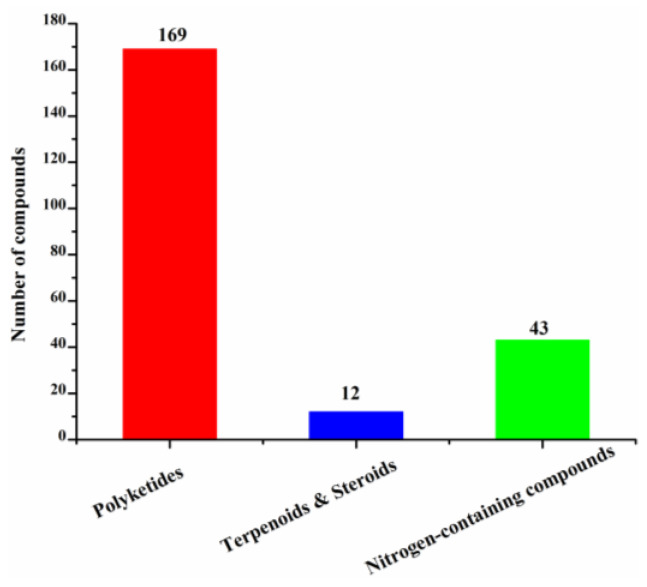
Structural classes of new halogenated compounds (1994–2019).

**Figure 13:**
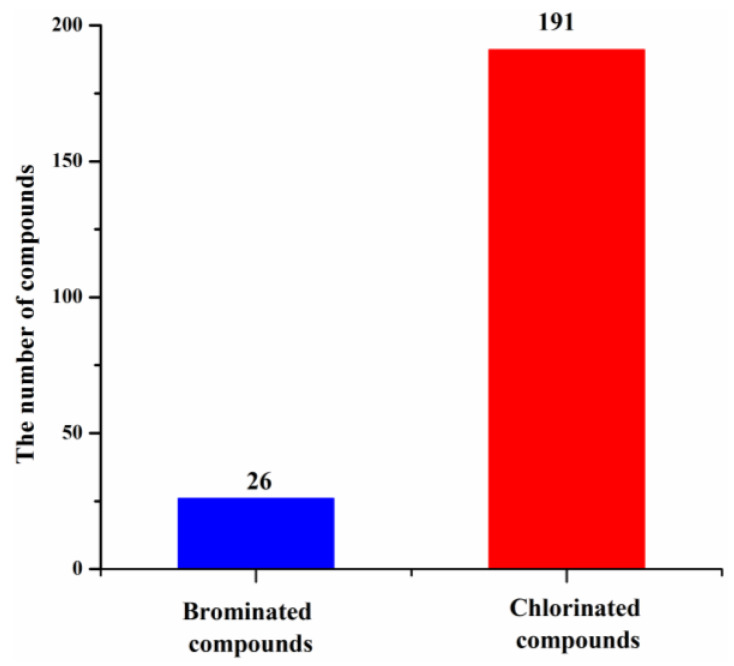
Proportion of new halogenated compounds (1994–2019).

These new marine natural products from marine fungi have different structure skeletons including polyketides, nitrogen-containing compounds, sterols, and terpenoids (Figure 12). Polyketides account for the majority (169, 78%) of the new halogenated compounds (217) isolated from marine fungi (Figure 12). The number of chlorinated compounds is 191, which is far more than that of brominated compounds simply due to the fact that chloride/chlorine is dominant in the Ocean when compared with bromide/bromine (Figure 13).

One of the challenges of discovering promising biologically active secondary metabolites from marine fungi is to mimic the culture environment as the marine. The surrounding environment such as oxygen, pressure, light, and salinity etc. significantly influence the growth of the marine fungi, as well as their ability to produce secondary metabolites. Although it is a challenge, investigating marine fungi for their halogenated secondary metabolites is worth it since more than 60% halogenated compounds isolated from marine fungi have some kind of significant biological activities. It is also worthy to assess halogenated compounds in a broader range of assays.

## Figures and Tables

**Table 1:** The initial research on antimicrobial active compounds from fungi.

First Producing Strain	Environment Source	Compound	Time
*Penicillium Terrestre*	Sediment, Jiaozhou Bay, China	Terrestrols B, D, F–G, a monomer (**19**)	2008
*Aspergillus* cf. *ochraceus* 941026	*Jaspis of Coriacea*, Indian-Pacific Ocean	Chlorocarolides A–B (**39** and **40**)	1996
Unidentified fungus	Indo-Pacific sponge *Jaspis aff. johnstoni*	Chloriolins A–C (**74**–**76**)	1994

**Table 2:** Halogenated compounds isolated from marine fungi (1994–2019).

Compound	Producing Strain	Environment Source	Bioactivity	Ref.
**1–2**	*Penicillium sclertiorum* GDST-2013-0415	Unidentified sponge GDST-2013-04, the coral reef at a depth of 10 m in the sea area of Shantou, Guangdong, China	-	[5]
**3–5**	*Penicillium canescens* 4.14. 6a.	The inner tissues of the marine sponge *Agelas oroides*, the coast of Sığacı̧kİzmir, Turkey	-	[6]
**6–8**	*Penicillium* sp. SCS-KFD09	A marine worm, *Sipunculus nudus* (HK10404), Haikou Bay, China	**6**: Anti-H_1_N_1_ activity; **8**: Protein tyrosine phosphatase 1B inhibitory activity	[7,8]
**9–10**	*Penicillium chrysogenum*	*Hypnea complex*, South Gyeongsang, Korea	DPPH activity	[9]
**11–12**	*Penicillium janthinellum* HDN13-309	*Sonneratia caseolari*, Hainan, China	cytotoxicity	[10]
**13–14**	*Penicillium citrinum* HL-5126	*Bruguiera sexangula var. rhynchopetala*, the South China Sea	**14**: Antibacterial activity	[11]
**15–19**	*Penicillium Terrestre*	Sediment, Jiaozhou Bay, China	cytotoxicity; **15**: DPPH activity	[12]
**20–23**	*Penicillium Terrestre*	Sediment, Jiaozhou Bay, China	**20–21**: Cytotoxicity	[13]
**24–27**	*Penicillium* sp. PR19N-1	Sediment (−1000 m), Prydz Bay, Antarctica	**24**: Cytotoxicity	[14]
**28**	*Penicillium* CF07370	Sediment (~100 m), Bahia de Los Angeles (Gulf of California, Mexico)	cytotoxicity	[15]
**29–31**	*Penicillium* sp. SCSIO sof101	Sediment (2448 m), the South China Sea (112°124′ E, 18°0.541′ N)	**29**: Cytotoxicity	[16]
**32–35**	*Penicillium janthinellum* HK1-6	The mangrove rhizosphere soil, Dongzhaigang mangrove natural reserve, Hainan Island	antibacterial activity	[17,18]
**36**	*Penicillium canescentia* MMS351	Seawater, French Atlantic coast	cytotoxicity	[19,20]
**37**	*Penicillium copticola* TPU1270	Marine foam, Iriomote Island, Okinawa Prefecture, Japan	-	[21]
**38**	*Penicillium sclerotiorum* M-22	The rotten leaf sample, on the west coast of Haikou, Hainan, China	cytotoxicity, antibacterial activity	[22]
**39–40**	*Aspergillus* cf. *ochraceus* 941026	*Jaspis of Coriacea*, Indian-Pacific Ocean	-	[23]
**41–43**	*A. ostianus* TUF 01F313	Unidentified sponge, Pohnpei, Micronesia	antibacterial activity	[24]
**44–46**	*Aspergillus unguis* CRI282-03	Unidentified sponge CRI282, Thailand	**44–46**: Aromatase inhibitor **45**: DPPH	[25]
**47**	*Aspergillus* sp. OUCMDZ-1583	An unidentified marine sponge XD10410, Xisha Islands, China	α-glucosidase inhibitor	[26]
**48**	*Aspergillus flocculosus* 16D-1	The sponge Phakellia fusca, Yongxing Island, China	inhibitory activity towards THP-1 and NO production in LPS-activated RAW264.7	[27]
**49–50**	*Aspergillus* sp. MF297-2	*Mytilus edulis*, Japan	-	[28,29]
**51**	*Aspergillus* sp. SCSGAF0093	*Melitodes squamata* collected from the South China Sea	-	[30]
**52–53**	*Aspergillus* sp. KMM 4676	Unidentified colonial ascidian, Shikotan Island, Pacific Ocean	**52**: Cytotoxicity	[31]
**54–55**	*Aspergillus sydowii*	*Acanthophora spicifera*, Bay of Bengal India	-	[32]
**56**	*Aspergillus* sp. MFB024	*Sargassum horneri*, Korea	antibacterial activity	[33]
**57**	*Aspergillus ochraceus*	Marine red alga *Chondria crassicualis*, Yokji Island, Kyeongnam, Korea	DPPH activity	[34]
**58**	*Aspergillus nidulans* EN-330	Marine red alga *P. scopulorum* var. *villum*, Yantai, China	cytotoxicity, antibacterial activity	[35]
**59–61**	*Aspergillus alliaceus*	Marine alga by Bioviotica GmbH	cytotoxicity	[36]
**62** **–** **70**	*Aspergillus sp. SCSIO* F063	A marine sediment sample, the South China Sea	**63**, **64**, **70**: Cytotoxicity	[37]
**71**	*A. westerdijkiae* DFFSCS013	A marine sediment sample, the South China Sea	cytotoxicity	[38]
**72**	*Aspergillus* sp. AF119	Sediment, Xiamen beach, China	-	[39]
**73**	*Aspergillus ungui* NKH-007	Soil (331 m), Suruga Bay, Japan (138°18.1207’ E, 34°22.4813’ N)	inhibitor of sterol O-acyltransferase	[40]
**74–76**	unidentified fungus	Indo-Pacific sponge *Jaspis aff. johnstoni*	**74**: Cytotoxicity	[41]
**77–78**	*Trichoderma harzianum* OUPS-N115	*Halichondria okadai*, Japan	cytotoxicity	[42,43]
**79–93**	*Gymnascella dankaliensis*	*Halichondria japonica*, Japan	cytotoxicity	[44,45,46,47,48,49,50,51]
**94–95**	*Exophiala* sp.	Sponge *Halichondria panicea*, Bogil Island, Jeonnam Province, Korea	antibacterial activity	[52]
**96–98**	*Acremonium* sp. J05B-1-F-3	Sponge *Stelletta* sp. (J05B-1), the coast of Jeju Island, Korea	-	[53]
**99–101**	*Acremonium persicinum*	Sponge *Anomoianthella rubrawere*, the gneering reef offshore from Mooloolaba	-	[54]
**102–104**	*Bartalinia robillardoides* LF550	Marine sponge *Tethya aurantium*, the Limsky kanal (Canal di Lemme or Limsky channel, Croatia)	**104**: Antibacterial activity **103–104**: Inhibitory activity towards PDE4	[55]
**105**	*Talaromyces minioluteus*	Unidentified marine sponge, Pilae Bay, Phi Phi Island, Krabi Province, Thailand	cytotoxicity	[56]
**106**	*Stachybotry sp.* HH1 ZSDS1F1-2	Sponge, Xisha Island, China	anti-virus activity	[57]
**107**	*Phoma* sp. 135	Sponge *Ectyplasia perox*, Dominica	-	[58]
**108–109**	fungus LL-37H248	Orange coral, Dixon Bay, Vancouver Island, Canada	**108**: Cytotoxicity	[59,60]
**110**	*Cochliobolus lunatus*	Gorgonian *Dichotella gemmacea*, the South China Sea	-	[61,62]
**111**	*Chondrostereum* sp. nov. SF002	*Sarcophyton tortuosum*, Sanya, Hainan	antibacterial activity	[63]
**112**	*Pestalotiopsis* sp. ZJ-2009-7-6	*Sarcophyton* sp., Yongxing Island	antibacterial activity	[64,65]
**113**	*Emericella unguis* M87-2	*Stomolopus meliagris*, Paria Bay, Venezuela	antibacterial activity	[66]
**114–115**	*Acremonium striatisporum* KMM 4401	*Eupentacta fraudatrix*, Japan	-	[67]
**116–118**	*Periconia byssaides* OUPS-N133	Sea hare *Aplysia kurodai*, Japan	-	[68,69,70]
**119–140**	*Chaetomium globosum* OUPS-T106B-6	Marine fish *Mugil cephalus*, Japan	**119**–**128**, **130**–**131**, **133**: Cytotoxicity	[71,72,73,74,75,76,77,78]
**141–144**	*Malbranchea graminicola* 086937A	Unidentified invertebrate, Kona, Hawaii	-	[79]
**145–146**	*Cochliobolus lunatus* TA26-46	*Palythoa haddoni*, Weizhou Island	-	[80]
**147**	*Trichoderma virens* CNL910	*Didemnum mole,* Papua New Guinea	cytotoxicity, antimicrobial activity	[81,82]
**148**	*Roussoella* sp. DLM33	the ascidian *Didemnum ligulum*, the north coast of São Paulo state, Brazil	-	[83]
**149–150**	*Pseudallescheria boydii*	*Acanthaster planci*, Hainan Sanya National Coral Reef Reserve, Hainan	-	[84]
**151**	*Pestalotia* sp. CNL-365	*Rosenvingea* sp. Bahamas	cytotoxicity, antimicrobial activity	[85,86]
**152–153**	unidentified fungus	*Gracillaria verrucose*, Korea	-	[87]
**154**	*Beauveria felina*	*Caulerpa* sp., São Paulo	-	[88]
**155**	*Enteromorpha compressa*, Busan, Korea	*Botrytis* sp.	-	[89]
**156**	*Acremonium* sp.	*Plocamium* sp., Heligoland	-	[90]
**157**	*Chaetomium* sp.	The algal species (taxonomy not determined), Kamari on the island Santorini, Greece	antiprotozoal activities	[91]
**158**	*Curvularia* sp. 768	*Acanthophora spicifera*, The Territory of Guam	-	[92]
**159–160**	*Fusarium tricinctum*	*Sargassum ringgoldium*, Yeosu, Korea	antibacterial activity	[93]
**161–162**	*Phoma herbarum*	*Gloiopeitis tenax*, Korea	DPPH activity	[94]
**163**	*Trichoderma* sp. (*cf. T. brevicompactum*) TPU199	A red alga, Palau	-	[95]
**164**	*Trichoderma asperellum* cf44-2	Marine brown alga *Sargassum* sp., Zhoushan Islands	antibacterial activity	[96]
**165**	*Sporothrix* sp. 4335	The bark of an estuarine mangrove, the South China Sea	-	[97]
**166–167**	*Emericella* sp. HK-ZJ	*A. corniculatu*, Haikou, China	antiviral activity	[98]
**168–173**	*Pestalotiopsis* sp. PSU-MA69	*R. apiculate*, Thailand	-	[99]
**174**	*Acremonium strictum*	The mangrove tree *Rhizophora apiculate* Blume	-	[100]
**175**	*Paradictyoarthrinium diffractum* BCC 8704	A mangrove wood in Laem Son National Park, Ranong Province, Thailand	cytotoxicity	[101]
**176** **–** **177**	*Lasiodiplodia theobromae* ZJ-HQ1	The marine mangrove *A. ilicifolius,* China	cytotoxicity, antibacterial activity	[102]
**178–179**	*Mucor irregularis* QEN-189	Mangrove plant *Rhizophora stylosa*, Hainan Island, China	cytotoxicity	[103]
**180–183**	*Rhinocladiella similis*	*Acrostichums aureum* (Pteridaceae), Douala, Cameroon	**180**: Cytotoxicity	[104]
**184**	*Polyporales* sp. PSU-ES44	*Thalassia hemprichii*	-	[105]
**185**	marine-derived fungus FOM-8108	Marine sand, Katase Enoshima Beach, Kanagawa, Japan	nSMase activity	[106]
**186–199**	*Spiromastix* sp. MCCC3A00308	Deep-sea sediment (2869 m), the South Atlantic Ocean (GPS 13.7501 W, 15.1668 S)	antibacterial activity	[107]
**200**	*Emericella* sp. SCSIO 05240	A sediment sample (3258 m), the South China Sea	antibacterial activity	[108]
**201**	*Cladosporium cladosporioides* HDN14-342	Sediment sample, Indian Ocean	cytotoxicity	[109]
**202–208**	*Pestalotiopsis neglecta*	Marine sediment (−10 m), Gageo, Korea	**205**–**208**: Cytotoxicity	[110]
**209** **–** **211**	*Chaetomium globosum* HDN151398	The sediment sample, South China Sea	**211**: Cytotoxicity	[111]
**212–213**	*F. heterosporum* CNC-477	A driftwood sample, Sweetings Cay, Bahamas	**212**: Cytotoxicity; **213**: Antibacterial activity	[112]
**214** **–** **217**	*Chaetomium* sp. NA-S01-R1	A seawater sample, the West Pacific Ocean	antimicrobial activity, cytotoxicity	[113]

## Data Availability

The data presented in this study are available in this article and supplementary material.

## Supplementary Materials

The Supplementary Materials are available online.

## Sample Availability

Samples of the compounds are not available from the authors.

## References

[1] Zhao C., Zhu T., Zhu W. (2013). New marine natural products of microbial origin from 2010 to 2013. Chin. J. Org. Chem..

[2] Butler A., Sandy M. (2009). Mechanistic considerations of halogenating enzymes. Nature.

[3] Ballschmiter K. (2003). Review: Pattern and sources of naturally produced organohalogens in the marine environment: Biogenic formation of organohalogens. Chemosphere.

[4] Gribble G.-W. (2003). The diversity of naturally produced organohalogens. Chemosphere.

[5] Wang C.-Y., Hao J.-D., Ning X.-Y., Wu J.-S., Zhao D.-L., Kong C.-J., Shao C.-L., Wang C.-Y. (2018). Penicilazaphilones D and E: Two new azaphilones from a sponge-derived strain of the fungus *Penicillium sclerotiorum*. RSC Adv..

[6] Frank M., Hartmann R., Plenker M., Mándi A., Kurtán T., Özkaya F.-C., Müller W.-E., Kassack M.-U., Hamacher A., Lin W. (2019). Brominated azaphilones from the sponge-associated fungus *Penicillium canescens* strain 4.14. 6a. J. Nat. Prod..

[7] Kong F.-D., Ma Q.-Y., Huang S.-Z., Wang P., Wang J.-F., Zhou L.-M., Yuan J.-Z., Dai H.-F., Zhao Y.-X. (2017). Chrodrimanins K–N and related meroterpenoids from the fungus *Penicillium* sp. SCS-KFD09 isolated from a marine worm, *Sipunculus nudus*. J. Nat. Prod..

[8] Kong F.-D., Zhang R.-S., Ma Q.-Y., Xie Q.-Y., Wang P., Chen P.-W., Zhou L.-M., Dai H.-F., Luo D.-Q., Zhao Y.-X. (2017). Chrodrimanins O–S from the fungus *Penicillium* sp. SCS-KFD09 isolated from a marine worm, *Sipunculus nudus*. Fitoterapia.

[9] Yang G., Yun K., Nenkep V.-N., Choi H.-D., Kang J.-S., Son B.-W. (2010). Induced production of halogenated diphenyl ethers from the marine-derived fungus *Penicillium chrysogenum*. Chem. Biodivers..

[10] Zhu M., Zhang X., Feng H., Dai J., Li J., Che Q., Gu Q., Zhu T., Li D. (2017). Penicisulfuranols A–F, alkaloids from the mangrove endophytic fungus *Penicillium janthinellum* HDN13-309. J. Nat. Prod..

[11] He K.-Y., Zhang C., Duan Y.-R., Huang G.-L., Yang C.-Y., Lu X.-R., Zheng C.-J., Chen G.-Y. (2017). New chlorinated xanthone and anthraquinone produced by a mangrove-derived fungus *Penicillium citrinum* HL-5126. J. Antibiot..

[12] Chen L., Fang Y., Zhu T., Gu Q., Zhu W. (2008). Gentisyl alcohol derivatives from the marine-derived fungus *Penicillium Terrestre*. J. Nat. Prod..

[13] Li D., Chen L., Zhu T., Kurtán T., Mándi A., Zhao Z., Li J., Gu Q. (2011). Chloctanspirones A and B, novel chlorinated polyketides with an unprecedented skeleton, from marine sediment derived fungus *Penicillium Terrestre*. Tetrahedron.

[14] Wu G., Lin A., Gu Q., Zhu T., Li D. (2013). Four new chloro-eremophilane sesquiterpenes from an Antarctic deep-sea derived fungus, *Penicillium* sp. PR19N-1. Mar. Drugs.

[15] Cardoso-Martínez F., José M., Díaz-Marrero A.-R., Darias J., Cerella C., Diederich M., Cueto M. (2015). Tanzawaic acids isolated from a marine-derived fungus of the genus *Penicillium* with cytotoxic activities. Org. Biomol. Chem..

[16] Luo M., Cui Z., Huang H., Song X., Sun A., Dang Y., Lu L., Ju J. (2017). Amino acid conjugated anthraquinones from the marine-derived fungus *Penicillium* sp. SCSIO sof101. J. Nat. Prod..

[17] Chen M., Shen N.-X., Chen Z.-Q., Zhang F.-M., Chen Y. (2017). Penicilones A–D, anti-MRSA azaphilones from the marine-derived fungus *Penicillium janthinellum* HK1-6. J. Nat. Prod..

[18] Chen M., Zheng Y.-Y., Chen Z.-Q., Shen N.-X., Shen L., Zhang F.-M., Zhou X.-J., Wang C.-Y. (2019). NaBr-induced production of brominated azaphilones and related tricyclic polyketides by the marine-derived fungus *Penicillium janthinellum* HK1-6. J. Nat. Prod..

[19] Vansteelandt M., Blanchet E., Egorov M., Petit F., Toupet L., Bondon A., Monteau F., Le Bizec B., Thomas O.-P., Pouchus Y.-F. (2013). Ligerin, an antiproliferative chlorinated sesquiterpenoid from a marine-derived *Penicillium* strain. J. Nat. Prod..

[20] Blanchet E., Vansteelandt M., Le Bot R., Egorov M., Guitton Y., Pouchus Y.-F., Grovel O. (2014). Synthesis and antiproliferative activity of ligerin and new fumagillin analogs against osteosarcoma. Eur. J. Med. Chem..

[21] Bu Y.-Y., Yamazaki H., Ukai K., Namikoshi M. (2015). Penicillimide, an open-chain hemisuccinimide from Okinawan marine-derived *Penicillium copticola*. J. Antibiot..

[22] Zhou S.-L., Wang M., Zhao H.-G., Huang Y.-H., Lin Y.-Y., Tan G.-H., Chen S.-L. (2016). Penicilazaphilone C, a new antineoplastic and antibacterial azaphilone from the marine fungus *Penicillium sclerotiorum*. Arch. Pharm. Res..

[23] Abrell L.-M., Borgeson B., Crews P. (1996). Chloro polyketides from the cultured fungus (*Aspergillus*) separated from a marine sponge. Tetrahedron Lett..

[24] Namikoshi M., Negishi R., Nagai H., Dmitrenok A., Kobayashi H. (2003). Three new chlorine containing antibiotics from a marine-derived fungus *Aspergillus ostianus* collected in Pohnpei. J. Antibiot..

[25] Sureram S., Wiyakrutta S., Ngamrojanavanich N., Mahidol C., Ruchirawat S., Kittakoop P. (2012). Depsidones, Aromatase inhibitors and radical scavenging agents from the marine-derived fungus *Aspergillus unguis* CRI282-03. Planta Med..

[26] Kong F., Zhao C., Hao J., Wang C., Wang W., Huang X., Zhu W. (2015). New α-glucosidase inhibitors from a marine sponge-derived fungus, *Aspergillus* sp. OUCMDZ-1583. RSC Adv..

[27] Gu B.-B., Jiao F.-R., Wu W., Liu L., Jiao W.-H., Sun F., Wang S.-P., Yang F., Lin H.-W. (2019). Ochrasperfloroid, an ochratoxin–ergosteroid heterodimer with inhibition of IL-6 and NO production from *Aspergillus flocculosus* 16D-1. RSC Adv..

[28] Tsukamoto S., Kawabata T., Kato H., Greshock T.-J., Hirota H., Ohta T., Williams R.-M. (2009). Isolation of antipodal (−)-versicolamide B and notoamides L–N from a marine-derived *Aspergillus* sp.. Org. Lett..

[29] Tsukamoto S., Umaoka H., Yoshikawa K., Ikeda T., Hirota H. (2010). Notoamide O, a structurally unprecedented prenylated indole alkaloid, and notoamides P-R from a marine-derived fungus, *Aspergillus* sp.. J. Nat. Prod..

[30] Xu X., He F., Zhang X., Bao J., Qi S. (2013). New mycotoxins from marine-derived fungus *Aspergillus* sp. SCSGAF0093. Food Chem. Toxicol..

[31] Ivanets E.-V., Yurchenko A.-N., Smetanina O.-F., Rasin A.-B., Zhuravleva O.-I., Pivkin M.-V., Popov R.-S., Von Amsberg G., Afiyatullov S.-S., Dyshlovoy S.-A. (2018). Asperindoles A–D and a p-terphenyl derivative from the ascidian-derived fungus *Aspergillus* sp. KMM 4676. Mar. Drugs.

[32] Teuscher F., Lin W., Wray V., Edrada R., Padmakumar K., Proksch P., Ebel R. (2006). Two new cyclopentanoids from the endophytic fungus *Aspergillus sydowii* associated with the marine alga *Acanthophora spicifera*. Nat. Prod. Commun..

[33] Nguyen H.-P., Zhang D., Lee U., Kang J.-S., Choi H.-D., Son B.-W. (2007). Dehydroxychlorofusarielin B, an antibacterial polyoxygenated decalin derivative from the marine-derived fungus *Aspergillus* sp.. J. Nat. Prod..

[34] Yun K., Feng Z., Choi H.-D., Kang J.-S., Son B.-W. (2013). New production of (*R*)-(–)-5-bromomellein, a dihydroisocoumarin derivative from the marine-derived fungus *Aspergillus ochraceus*. Chem. Nat. Compd..

[35] Zhang P., Li X.-M., Li X., Wang B.-G. (2015). New indole-diterpenoids from the algal-associated fungus *Aspergillus nidulans*. Phytochem. Lett..

[36] Mandelare P.-E., Adpressa D.-A., Kaweesa E.-N., Zakharov L.-N., Loesgen S. (2018). Coculture of two developmental stages of a marine-derived *Aspergillus alliaceus* results in the production of the cytotoxic bianthrone allianthrone A. J. Nat. Prod..

[37] Huang H., Wang F., Luo M., Chen Y., Song Y., Zhang W., Zhang S., Ju J. (2012). Halogenated anthraquinones from the marine-derived fungus *Aspergillus* sp. SCSIO F063. J. Nat. Prod..

[38] Peng J., Zhang X.-Y., Tu Z.-C., Xu X.-Y., Qi S.-H. (2013). Alkaloids from the deep-sea-derived fungus *Aspergillus westerdijkiae* DFFSCS013. J. Nat. Prod..

[39] Liu S., Lu C., Huang J., Shen Y. (2012). Three new compounds from the marine fungal strain *Aspergillus* sp. AF119. Rec. Nat. Prod..

[40] Uchida R., Nakajyo K., Kobayashi K., Ohshiro T., Terahara T., Imada C., Tomoda H. (2016). 7-Chlorofolipastatin, an inhibitor of sterol O-acyltransferase, produced by marine-derived *Aspergillus ungui* NKH-007. J. Antibiot..

[41] Cheng X.-C., Varoglu M., Abrell L., Crews P., Lobkovsky E., Clardy J. (1994). Chloriolins A-C, chlorinated sesquiterpenes produced by fungal cultures separated from a *Jaspis* marine sponge. J. Org. Chem..

[42] Amagata T., Usami Y., Minoura K., Ito T., Numata A. (1998). Cytotoxic substances produced by a fungal strain from a sponge: Physico-chemical properties and structures. J. Antibiot..

[43] Usami Y., Ikura T., Amagata T., Numata A. (2000). First total syntheses and configurational assignments of cytotoxic trichodenones A–C. Tetrahedron: Asymmetry.

[44] Numata A., Amagata T., Minoura K., Ito T. (1997). Gymnastatins, novel cytotoxic metabolites produced by a fungal strain from a sponge. Tetrahedron Lett..

[45] Amagata T., Doi M., Ohta T., Minoura K., Ito T., Numata A. (1998). Absolute stereostructures of novel cytotoxic metabolites, gymnastatins A-E, from a *Gymnascella* species separated from a *Halichondria* sponge. J. Chem. Soc. Perkin Trans. 1.

[46] Amagata T., Minoura K., Numata A. (2006). Gymnastatins F-H, cytostatic metabolites from the sponge-derived fungus *Gymnascella dankaliensis*. J. Nat. Prod..

[47] Amagata T., Takigawa K., Minoura K. (2010). Gymnastatins I-K, Cancer cell growth inhibitors from a sponge-derived *Gymnascella dankaliensis*. Heterocycles.

[48] Amagata T., Tanaka M., Yamada T., Minoura K., Numata A. (2008). Gymnastatins and dankastatins, growth inhibitory metabolites of a *Gymnascella* species from a *Halichondria* Sponge. J. Nat. Prod..

[49] Amagata T., Tanaka M., Yamada T., Chen Y.-P., Minoura K., Numata A. (2013). Additional cytotoxic substances isolated from the sponge-derived *Gymnascella dankaliensis*. Tetrahedron Lett..

[50] Ogamino T., Ohnishi S., Ishikawa Y., Sugai T., Obata R., Nishiyama S. (2006). Synthesis and biological assessment of hemiacetal spiro derivatives towards development of efficient chemotherapeutic agent. Sci. Technol. Adv. Mater..

[51] Murayama K., Tanabe T., Ishikawa Y., Nakamura K., Nishiyama S. (2009). A synthetic study on gymnastatins F and Q: The tandem Michael and aldol reaction approach. Tetrahedron Lett..

[52] Zhang D., Yang X., Kang J.-S., Choi H.-D., Son B.-W. (2008). Chlorohydroaspyrones A and B, antibacterial aspyrone derivatives from the marine-derived fungus *Exophiala* sp.. J. Nat. Prod..

[53] Zhang P., Bao B., Dang H.-T., Hong J., Lee H.-J., Yoo E.-S., Bae K.-S., Jung J.-H. (2009). Anti-inflammatory sesquiterpenoids from a sponge-derived fungus *Acremonium* sp.. J. Nat. Prod..

[54] Fraser J.-A., Lambert L.-K., Pierens G.-K., Bernhardt P.-V., Garson M.-J. (2013). Secondary metabolites of the sponge-derived fungus *Acremonium persicinum*. J. Nat. Prod..

[55] Jansen N., Ohlendorf B., Erhard A., Bruhn T., Bringmann G., Imhoff J.-F. (2013). Helicusin E, isochromophilone X and isochromophilone XI: New chloroazaphilones produced by the fungus *Bartalinia robillardoides* Strain LF550. Mar. Drugs.

[56] Ngokpol S., Suwakulsiri W., Sureram S., Lirdprapamongkol K., Aree T., Wiyakrutta S., Mahidol C., Ruchirawat S., Kittakoop P. (2015). Drimane sesquiterpene-conjugated amino acids from a marine isolate of the fungus *Talaromyces minioluteus* (*Penicillium minioluteum*). Mar. Drugs.

[57] Qin C., Lin X., Lu X., Wan J., Zhou X., Liao S., Tu Z., Xu S., Liu Y. (2015). Sesquiterpenoids and xanthones derivatives produced by sponge-derived fungus *Stachybotry* sp. HH1 ZSDS1F1-2. J. Antibiot..

[58] Elsebai M.-F., Ghabbour H.-A. (2016). Isocoumarin derivatives from the marine-derived fungus *Phoma* sp. 135. Tetrahedron Lett..

[59] McDonald L.-A., Abbanat D.-R., Barbieri L.-R., Bernan V.-S., Discafani C.-M., Greenstein M., Janota K., Korshalla J.-D., Lassota P., Tischler M. (1999). Spiroxins, DNA cleaving antitumor antibiotics from a marine-derived fungus. Tetrahedron Lett..

[60] Ando Y., Tanaka D., Sasaki R., Ohmori K., Suzuki K. (2019). Stereochemical dichotomy in two competing cascade processes: Total syntheses of both enantiomers of spiroxin A. Angew. Chem..

[61] Shao C.-L., Wu H.-X., Wang C.-Y., Liu Q.-A., Xu Y., Wei M.-Y., Qian P.-Y., Gu Y.-C., Zheng C.-J., She Z.-G. (2011). Potent antifouling resorcylic acid lactones from the gorgonian-derived fungus *Cochliobolus lunatus*. J. Nat. Prod..

[62] Mahankali B., Srihari P.-A. (2015). Carbohydrate approach for the first total synthesis of cochliomycin C: Stereoselective total synthesis of paecilomycin E, paecilomycin F and 6′-epi-Cochliomycin, C. Eur. J. Org. Chem..

[63] Li H.-J., Chen T., Xie Y.-L., Chen W.-D., Zhu X.-F., Lan W.-J. (2013). Isolation and structural elucidation of chondrosterins F-H from the marine fungus *Chondrostereum* sp.. Mar. Drugs.

[64] Wei M.-Y., Li D., Shao C.-L., Deng D.-S., Wang C.-Y. (2013). (±)-Pestalachloride D, an antibacterial racemate of chlorinated benzophenone derivative from a soft coral-derived fungus *Pestalotiopsis* sp.. Mar. Drugs.

[65] Arredondo V., Roa D.-E., Yan S., Liu-Smith F., Van Vranken D.-L. (2019). Total synthesis of (±)-pestalachloride C and (±)-pestalachloride D through a biomimetic knoevenagel/hetero-diels–alder cascade. Org. Lett..

[66] Nielsen J., Nielsen P.-H., Frisvad J.-C. (1999). Fungal depside, guisinol, from a marine derived strain of *Emericella unguis*. Phytochemistry.

[67] Afiyatullov S.-S., Kalinovsky A.-I., Antonov A.-S. (2011). New virescenosides from the marine-derived fungus *Acremonium striatisporum*. Nat. Prod. Commun..

[68] Yamada T., Iritani M., Ohishi H., Tanaka K., Minoura K., Doi M., Numata A. (2007). Pericosines, antitumour metabolites from the sea hare-derived fungus *Periconia byssoides*. Structures and biological activities. Org. Biomol. Chem..

[69] Boyd D.-R., Sharma N.-D., Acaru C.-A., Malone J.-F., O’Dowd C.-R., Allen C.-C., Stevenson P.-J. (2010). Chemoenzymatic synthesis of carbasugars (+)-pericosines A–C from diverse aromatic cis-dihydrodiol precursors. Org. Lett..

[70] Mizuki K., Iwahashi K., Murata N., Ikeda M., Nakai Y., Yoneyama H., Harusawa S., Usami Y. (2014). Synthesis of marine natural product (−)-pericosine E. Org. Lett..

[71] Yamada T., Doi M., Shigeta H., Muroga Y., Hosoe S., Numata A., Tanaka R. (2008). Absolute stereostructures of cytotoxic metabolites, chaetomugilins A-C, produced by a *Chaetomium* species separated from a marine fish. Tetrahedron Lett..

[72] Yasuhide M., Yamada T., Numata A., Tanaka R. (2008). Chaetomugilins, new selectively cytotoxic metabolites, produced by a marine fish-derived *Chaetomium* species. J. Antibiot..

[73] Yamada T., Yasuhide M., Shigeta H., Numata A., Tanaka R. (2009). Absolute stereostructures of chaetomugilins G and H produced by a marine-fish-derived *Chaetomium* species. J. Antibiot..

[74] Muroga Y., Yamada T., Numata A., Tanaka R. (2009). Chaetomugilins I-O, new potent cytotoxic metabolites from a marine-fish-derived *Chaetomium* species. Stereochemistry and biological activities. Tetrahedron.

[75] Yamada T., Muroga Y., Tanaka R. (2009). New azaphilones, seco-chaetomugilins A and D, produced by a marine-fish-derived *Chaetomium globosum*. Mar. Drugs.

[76] Muroga Y., Yamada T., Numata A., Tanaka R. (2010). 11- and 4′-Epimers of chaetomugilin A, novel cytostatic metabolites from marine fish-derived fungus *Chaetomium globosum*. Helv. Chim. Acta.

[77] Yamada T., Muroga Y., Jinno M., Kajimoto T., Usami Y., Numata A., Tanaka R. (2011). New class azaphilone produced by a marine fish-derived *Chaetomium globosum*. The stereochemistry and biological activities. Bioorg. Med. Chem..

[78] Yamada T., Jinno M., Kikuchi T., Kajimoto T., Numata A., Tanaka R. (2012). Three new azaphilones produced by a marine fish-derived *chaetomium globosum*. J. Antibiot..

[79] Watts K.-R., Loveridge S.-T., Tenney K., Media J., Valeriote F.-A., Crews P. (2011). Utilizing DART Mass Spectrometry to pinpoint halogenated metabolites from a marine invertebrate-derived fungus. J. Org. Chem..

[80] Zhang W., Shao C.-L., Chen M., Liu Q.-A., Wang C.-Y. (2014). Brominated resorcylic acid lactones from the marine-derived fungus *Cochliobolus lunatus* induced by histone deacetylase inhibitors. Tetrahedron Lett..

[81] Garo E., Starks C.-M., Jensen P.-R., Fenical W., Lobkovsky E., Clardy J. (2003). Trichodermamides A and B, cytotoxic modified dipeptides from the marine-derived fungus *Trichoderma virens*. J. Nat. Prod..

[82] Lu C.-D., Zakarian A. (2008). Total synthesis of (±)-Trichodermamide B and of a putative biosynthetic precursor to Aspergillazine A using an oxaza-cope rearrangement. Angew. Chem. Int. Ed..

[83] Ferreira E.-L., Williams D.-E., Ioca L.-P., Morais-Urano R.-P., Santos M.-F., Patrick B.-O., Elias L.-M., Lira S.-P., Ferreira A.-G., Passarini M.-R. (2015). Structure and biogenesis of roussoellatide, a dichlorinated polyketide from the marine-derived fungus *Roussoella* sp. DLM33. Org. Lett..

[84] Yan D.-F., Lan W.-J., Wang K.-T., Huang L., Jiang C.-W., Li H.-J. (2015). Two chlorinated benzofuran derivatives from the marine fungus *Pseudallescheria boydii*. Nat. Prod. Commun..

[85] Cueto M., Jensen P.-R., Kauffman C., Fenical W., Lobkovsky E., Clardy J. (2001). Pestalone, a new antibiotic produced by a marine fungus in response to bacterial challenge. J. Nat. Prod..

[86] Slavov N., Cvengroš J., Neudörfl J.-M., Schmalz H.-G. (2010). Total synthesis of the marine antibiotic pestalone and its surprisingly facile conversion into pestalalactone and pestalachloride A. Angew. Chem. Int. Ed..

[87] Li X., Kim S.-K., Kang J.-S., Choi H.-D., Son B.-W. (2004). Polyketide and sesquiterpenediol metabolites from a marine-derived fungus. Bull. Korean Chem. Soc..

[88] Lira S.-P., Vita-Marques A.-M., Seleghim M.-H.-R., Bugni T.-S., LaBarbera D.-V., Sette L.-D., Sponchiado S.-R., Ireland C.-M., Berlinck R.-G. (2006). New destruxins from the marine-derived fungus *Beauveria felina*. J. Antibiot..

[89] Li X., Zhang D., Lee U., Li X., Cheng J., Zhu W., Jung J.-H., Choi H.-D., Son B.-W. (2007). Bromomyrothenone B and botrytinone, cyclopentenone derivatives from a marine isolate of the fungus *Botrytis*. J. Nat. Prod..

[90] Pontius A., Mohamed I., Krick A., Kehraus S., König G.-M. (2008). Aromatic polyketides from marine algicolous fungi. J. Nat. Prod..

[91] Pontius A., Krick A., Kehraus S., Brun R., König G.-M. (2008). Antiprotozoal activities of heterocyclic- substituted xanthones from the marine-derived fungus *Chaetomium* sp.. J. Nat. Prod..

[92] Greve H., Schupp P.-J., Eguereva E., Kehraus S., König G.-M. (2008). Ten-membered lactones from the marine-derived fungus *Curvularia* sp.. J. Nat. Prod..

[93] Nenkep V., Yun K., Zhang D., Choi H.-D., Kang J.-S., Son B.-W. (2010). Induced production of bromomethylchlamydosporols A and B from the marine-derived fungus *Fusarium tricinctum*. J. Nat. Prod..

[94] Nenkep V.-N., Yun K., Li Y., Choi H.-D., Kang J.-S., Son B.-W. (2010). New production of haloquinones, bromochlorogentisylquinones A and B, by a halide salt from a marine isolate of the fungus *Phoma herbarum*. J. Antibiot..

[95] Yamazaki H., Takahashi O., Murakami K., Namikoshi M. (2015). Induced production of a new unprecedented epitrithiodiketopiperazine, chlorotrithiobrevamide, by a culture of the marine-derived *Trichoderma* cf. *brevicompactum* with dimethyl sulfoxide. Tetrahedron Lett..

[96] Song Y.-P., Miao F.-P., Fang S.-T., Yin X.-L., Ji N.-Y. (2018). Halogenated and nonhalogenated metabolites from the marine-alga-endophytic fungus *Trichoderma asperellum* cf44-2. Mar. Drugs.

[97] Wen L., Guo Z.-Y., Li Q., Zhang D., She Z., Vrijmoed L.-L. (2010). A new griseofulvin derivative from the mangrove endophytic fungus *Sporothrix* sp.. Chem. Nat. Compd..

[98] Zhang G., Sun S., Zhu T., Lin Z., Gu J., Li D., Gu Q. (2011). Antiviral isoindolone derivatives from an endophytic fungus *Emericella* sp. associated with *Aegiceras corniculatum*. Phytochemistry.

[99] Klaiklay S., Rukachaisirikul V., Tadpetch K., Sukpondma Y., Phongpaichit S., Buatong J., Sakayaroj J. (2012). Chlorinated chromone and diphenyl ether derivatives from the mangrove-derived fungus *Pestalotiopsis* sp. PSU-MA69. Tetrahedron.

[100] Hammerschmidt L., Debbab A., Ngoc T.-D., Wray V., Hemphil C.-P., Lin W., Broetz-Oesterhelt H., Kassack M.-U., Proksch P., Aly A.-H. (2014). Polyketides from the mangrove-derived endophytic fungus *Acremonium strictum*. Tetrahedron Lett..

[101] Isaka M., Chinthanom P., Rachtawee P., Srichomthong K., Srikitikulchai P., Kongsaeree P., Prabpai S. (2015). Cytotoxic hydroanthraquinones from the mangrove-derived fungus *Paradictyoarthrinium diffractum* BCC 8704. J. Antibiot..

[102] Chen S., Chen D., Cai R., Cui H., Long Y., Lu Y., Li C., She Z. (2016). Cytotoxic and antibacterial preussomerins from the mangrove endophytic fungus *Lasiodiplodia theobromae* ZJ-HQ1. J. Nat. Prod..

[103] Gao S.-S., Li X.-M., Williams K., Proksch P., Ji N.-Y., Wang B.-G. (2016). Rhizovarins A–F, indole-diterpenes from the mangrove-derived endophytic fungus *Mucor irregularis* QEN-189. J. Nat. Prod..

[104] Liu S., Zhao Y., Heering C., Janiak C., Müller W.-E., Akoné S.-H., Liu Z., Proksch P. (2019). Sesquiterpenoids from the endophytic fungus *Rhinocladiella similis*. J. Nat. Prod..

[105] Rukachaisirikul V., Kannai S., Klaiklay S., Phongpaichit S., Sakayaroj J. (2013). Rare 2-phenylpyran-4-ones from the seagrass-derived fungi Polyporales PSU-ES44 and PSU-ES83. Tetrahedron.

[106] Uchida R., Tomoda H., Arai M., Omura S. (2001). Chlorogentisylquinone, a new neutral sphingo-myelinase inhibitor, produced by a marine fungus. J. Antibiot..

[107] Niu S., Liu D., Hu X., Proksch P., Shao Z., Lin W. (2014). Spiromastixones A-O, antibacterial chlorodepsidones from a deep-sea-derived *Spiromastix* sp. fungus. J. Nat. Prod..

[108] Fredimoses M., Zhou X., Lin X., Tian X., Ai W., Wang J., Liao S., Liu J., Yang B., Yang X. (2014). New prenylxanthones from the deep-sea derived fungus *Emericella* sp. SCSIO 05240. Mar. Drugs.

[109] Zhang Z., He X., Liu C., Che Q., Zhu T., Gu Q., Li D. (2016). Clindanones A and B and cladosporols F and G, polyketides from the deep-sea derived fungus *Cladosporium cladosporioides* HDN14-342. RSC Adv..

[110] Wang W., Park C., Oh E., Sung Y., Lee J., Park K.-H., Kang H. (2019). Benzophenone Compounds, from a Marine-Derived Strain of the Fungus *Pestalotiopsis neglecta*, Inhibit Proliferation of Pancreatic Cancer Cells by Targeting the MEK/ERK Pathway. J. Nat. Prod..

[111] Sun C., Ge X., Mudassir S., Zhou L., Yu G., Che Q., Zhang G., Peng J., Gu Q., Zhu T. (2019). New glutamine-containing azaphilone alkaloids from deep-sea-derived fungus *Chaetomium globosum* HDN151398. Mar. Drugs.

[112] Renner M.-K., Jensen P.-R., Fenical W. (1998). Neomangicols: Structures and absolute stereochemistries of unprecedented halogenated sesterterpenes from a marine fungus of the genus *Fusarium*. J. Org. Chem..

[113] Wang W., Liao Y., Chen R., Hou Y., Ke W., Zhang B., Gao M., Shao Z., Chen J., Li F. (2018). Chlorinated azaphilone pigments with antimicrobial and cytotoxic activities isolated from the deep sea derived fungus *Chaetomium* sp. NA-S01-R1. Mar. Drugs.

