# Supplementary material for: A Review: Halogenated Compounds from Marine Fungi
Source: Molecules. 2021 Jan 16;26(2):458. doi: 10.3390/molecules26020458 (PMC7830638; doi:10.3390/molecules26020458)
Supplement: Supplementary file 1 [file molecules-26-00458-s001.pdf]

Supplementary Materials

A Review of Halogenated Compounds from Marine Fungi

Cong Wang [1,2,*], Huanyun Lu [1], Jian Zhou Lan [1], KH Ahammad Uz Zaman [2] and Shugeng Cao [2,*]

[1] Key Laboratory of Chemistry and Engineering of Forest Products, State Ethnic Affairs Commission， Guangxi Key Laboratory of Chemistry and Engineering of Forest Products, Guangxi Collaborative Innovation Center for Chemistry and Engineering of Forest Products, School of Chemistry and Chemical Engineering, Guangxi University for Nationalities, Nanning 530006, China; wangcong123206@163.com (C.W.); luhuanyun2020@163.com (H.L.); lanjianzhou1576@163.com (J.L.)

[2] Department of Pharmaceutical Sciences, Daniel K. Inouye College of Pharmacy, University of Hawai'i at Hilo, Hilo, Hawaii 96720, United States; kzaman@hawaii.edu (K.A.Z.); scao@hawaii.edu (S.C.)

[*] Correspondences: scao@hawaii.edu; wangcong123206@163.com

Table S1. Chemical Formula of Halogenated compounds isolated from marine fungi (1994–2019)

The number of the compounds	The name of the compounds	Chemical Formula	The number of the compounds	The name of the compounds	Chemical Formula
**1**	Penicilazaphilone D	$C_{14}H_{15}ClO_5$	**110**	Cochliomycin C	$C_{19}H_{25}ClO_7$
**2**	Penicilazaphilone E	$C_{19}H_{25}ClO_6$	**111**	Chondrosterin H	$C_{10}H_9ClO_2$
**3**	Methyl 3-chloro-2-(2,4-dimethoxy-6-methylphenoxy)-6-hydroxy-4-methoxybenzoate	$C_{19}H_{21}ClO_6$	**113**	(±)-pestalachloride D	$C_{21}H_{20}Cl_2O_5$
**4**	Bromophilone A	$C_{33}H_{35}BrO_{13}$	**113**	Guisinol	$C_{23}H_{25}ClO_5$
**5**	Bromophilone B	$C_{33}H_{35}BrO_{13}$	**114**	Virescenoside Z_5	$C_{26}H_{39}ClO_9$
**6**	Chrodrimanin K	$C_{25}H_{31}ClO_6$	**115**	Virescenoside Z_7	$C_{26}H_{41}ClO_{10}$
**7**	Chrodrimanin L	$C_{25}H_{30}Cl_2O_6$	**116**	Pericosine A	$C_8H_{11}ClO_5$
**8**	Chrodrimanin O	$C_{25}H_{29}Cl_3O_6$	**117**	Pericosine D	$C_8H_{11}ClO_5$
**9**	4,6,4′,6′-tetrabromo-3,3′-dihydroxy-5,5′-dimethyldiphenyl ether	$C_{14}H_{10}Br_4O_3$	**118**	Pericosine E	$C_{16}H_{21}ClO_{10}$
**10**	4,6,2′,4′,6′-pentabromo-3,3′-dihydroxy-5,5′-dimethyldiphenyl ether	$C_{14}H_9Br_5O_3$	**119**	Chaetomugilin C	$C_{23}H_{25}ClO_6$
**11**	Penicisulfuranol A	$C_{21}H_{21}ClN_2O_8S_2$	**120**	Chaetomugilin D	$C_{23}H_{27}ClO_6$
**12**	Penicisulfuranol D	$C_{23}H_{27}ClN_2O_8S_2$	**121**	Chaetomugilin E	$C_{24}H_{29}ClO_6$
**13**	4-Chloro-1-hydroxy-3-	$C_{17}H_{13}ClO_6$	**122**	Chaetomugilin F	$C_{23}H_{25}ClO_5$

	methoxy-6-methyl-8-methoxycarbonyl-xanthen-9-one				
**14**	2′-acetoxy-7-chlorocitreorosein	$C_{18}H_{13}ClO_7$	**123**	Chaetomugilin G	$C_{24}H_{29}ClO_7$
**15**	Dimeric terrestrol B	$C_{14}H_{13}ClO_5$	**124**	Chaetomugilin H	$C_{24}H_{29}ClO_6$
**16**	Dimeric terrestrol D	$C_{14}H_{13}ClO_5$	**125**	Chaetomugilin I	$C_{22}H_{27}ClO_5$
**17**	Dimeric terrestrol F	$C_{14}H_{13}ClO_5$	**126**	Chaetomugilin J	$C_{22}H_{27}ClO_4$
**18**	Dimeric terrestrol G	$C_{14}H_{13}ClO_5$	**127**	Chaetomugilin K	$C_{23}H_{29}ClO_4$
**19**	Not named	$C_8H_9ClO_3$	**128**	Chaetomugilin L	$C_{23}H_{29}ClO_5$
**20**	Chloctanspirone A	$C_{21}H_{23}ClO_7$	**129**	Chaetomugilin M	$C_{24}H_{29}ClO_6$
**21**	Chloctanspirone B	$C_{21}H_{23}ClO_7$	**130**	Chaetomugilin N	$C_{23}H_{25}ClO_6$
**22**	Chloctanspirone K	$C_7H_9ClO_3$	**131**	Chaetomugilin O	$C_{23}H_{25}ClO_5$
**23**	Chloctanspirone L	$C_7H_9ClO_3$	**132**	*Seco*-chaetomugilin A	$C_{24}H_{31}ClO_8$
**24**	1-chloro-3β-acetoxy-7-hydroxy-trinoreremophil-1,6,9-trien-8-one	$C_{14}H_{15}ClO_4$	**133**	*Seco*-chaetomugilin D	$C_{24}H_{31}ClO_7$
**25**	Not named	$C_{15}H_{19}ClO_4$	**134**	11-*epi*-chaetomugilin A	$C_{23}H_{27}ClO_7$
**26**	1α-chloro-2β-hydroxyeremophil-7(11),9-dien-8-one	$C_{15}H_{21}ClO_2$	**135**	4′-*epi*-chaetomugilin A	$C_{23}H_{27}ClO_7$
**27**	Not named	$C_{18}H_{25}ClO_6$	**136**	Chaeto-mugilins P	$C_{22}H_{27}ClO_5$
**28**	Tanzawaic acid P	$C_{18}H_{27}ClO_4$	**137**	Chaeto-mugilins Q	$C_{22}H_{29}ClO_6$
**29**	Emodacidamide C	$C_{20}H_{16}ClNO_8$	**138**	Chaeto-mugilins R	$C_{16}H_{21}ClO_5$
**30**	Emodacidamide F	$C_{21}H_{18}ClNO_8$	**139**	11-*epi*-chaetomugilin I	$C_{22}H_{27}ClO_5$
**31**	Emodacidamide G	$C_{21}H_{18}ClNO_8$	**140**	Chaetomugilin S	$C_{23}H_{27}ClO_6$
**32**	Penicilone C	$C_{29}H_{35}ClO_7$	**141**	(−)-Spiromalbramide	$C_{21}H_{23}Cl_2N_3O_2$
**33**	Penicilone D	$C_{29}H_{33}ClO_6$	**142**	(+)-Isomalbrancheamide B	$C_{21}H_{24}ClN_3O$
**34**	Penicilone G	$C_{29}H_{35}BrO_7$	**143**	(+)-Malbrancheamide C	$C_{21}H_{24}BrN_3O$
**35**	Penicilone H	$C_{29}H_{33}BrO_6$	**144**	Isomalbrancheamide B	$C_{21}H_{24}BrN_3O$
**36**	Ligerin	$C_{20}H_{31}ClO_7$	**145**	5-bromozeaenol	$C_{19}H_{23}BrO_7$
**37**	Penicillimide	$C_{13}H_{14}ClNO_5$	**146**	3,5-dibromozeaenol	$C_{19}H_{22}Br_2O_7$
**38**	Penicilazaphilone C	$C_{20}H_{29}ClO_7$	**147**	Trichodermamide B	$C_{20}H_{19}ClN_2O_8$
**39**	Chlorocarolide A	$C_9H_{13}ClO_4$	**148**	Roussoellatide	$C_{19}H_{22}Cl_2O_6$
**40**	Chlorocarolide B	$C_9H_{13}ClO_4$	**149**	6-chloro-2-(2-hydroxypropan-2-yl)-2,3-dihydro-5-hydroxybenzofuran	$C_{11}H_{13}ClO_3$
**41**	8-chloro-9-hydroxy-8,9-deoxyasperlactone	$C_9H_{13}ClO_4$	**150**	7-chloro-2-(2-hydroxypropan-2-yl)-2,3-dihydro-5-hydroxybenzofuran	$C_{11}H_{13}ClO_3$
**42**	9-chloro-8-hydroxy-8,9-deoxyasperlactone	$C_9H_{13}ClO_4$	**151**	Pestalone	$C_{21}H_{20}Cl_2O_6$

**43**	9-chloro-8-hydroxy-8,9-deoxyaspyrone	$C_9H_{13}ClO_4$	**152**	Methyl 2,4-dibromo-5-oxo-2-decenoate	$C_{11}H_{16}Br_2O_3$
**44**	Aspergillusidone B	$C_{20}H_{18}Cl_2O_5$	**153**	Methyl 2,4-dibromo-5-oxo-3-decenoate	$C_{11}H_{16}Br_2O_3$
**45**	Aspergillusidone C	$C_{19}H_{16}Cl_2O_5$	**154**	[β-MePro] destruxin E	$C_{30}H_{50}ClN_5O_8$
**46**	Aspergillusether A	$C_{21}H_{21}Cl_3O_6$	**155**	Bromomyrothenone B	$C_7H_8BrNO_2$
**47**	Aspergone O	$C_{10}H_{15}ClO_4$	**156**	Acremonisol A	$C_{12}H_{15}ClO_4$
**48**	Ochrasperfloroid	$C_{48}H_{58}ClNO_{14}$	**157**	Chaetoxanthone C	$C_{20}H_{19}ClO_6$
**49**	Notoamide N	$C_{26}H_{28}ClN_3O_4$	**158**	91	$C_{10}H_{13}ClO_4$
**50**	Notoamide P	$C_{26}H_{30}BrN_3O_5$	**159**	Bromomethylchlamydosporol A	$C_{12}H_{15}BrO_5$
**51**	Ochratoxin A *n*-butyl ester	$C_{24}H_{26}ClNO_6$	**160**	Bromomethylchlamydosporol B	$C_{12}H_{14}Br_2O_5$
**52**	Asperindole A	$C_{29}H_{32}ClNO_6$	**161**	Bromochlorogentisylquinone A	$C_7H_4BrClO_3$
**53**	Asperindole C	$C_{33}H_{38}ClNO_8$	**162**	Bromochlorogentisylquinone B	$C_7H_4BrClO_3$
**54**	Sydowin A	$C_{18}H_{14}Cl_2O_7$	**163**	Chlorotrithiobrevamide	$C_{21}H_{23}ClN_2O_8S_3$
**55**	Sydowin B	$C_{18}H_{14}Cl_2O_6$	**164**	3-hydroxytrichodenone C	$C_7H_9ClO_3$
**56**	Dehydroxychlorofusarielin B	$C_{25}H_{39}ClO_4$	**165**	7-chloro-2′,5,6-trimethoxy-6′-methylspiro(benzofuran-2(3H),1′-(2) cyclohexene)3,4′-dione	$C_{17}H_{17}ClO_6$
**57**	(*R*)-(–)-5-bromomellein	$C_{10}H_9BrO_3$	**166**	Emeriphenolicin A	$C_{25}H_{36}ClNO_5$
**58**	19-hydroxypenitrem A	$C_{37}H_{44}ClNO_7$	**167**	Emeriphenolicin B	$C_{25}H_{36}ClNO_5$
**59**	Allianthrone A	$C_{36}H_{32}Cl_2O_{10}$	**168**	Pestalotether A	$C_{18}H_{17}ClO_8$
**60**	Allianthrone B	$C_{36}H_{32}Cl_2O_{10}$	**169**	Pestalotether B	$C_{16}H_{15}ClO_6$
**61**	Allianthrone C	$C_{36}H_{32}Cl_2O_{10}$	**170**	Pestalotether C	$C_{18}H_{17}ClO_8$
**62**	(1′*S*)-7-Chloroaverantin	$C_{20}H_{19}ClO_7$	**171**	Pestalochromone A	$C_{12}H_{17}ClO_5$
**63**	(1′*S*)-6-*O*-methyl-7-chloroaverantin	$C_{21}H_{21}ClO_7$	**172**	Pestalochromone B	$C_{12}H_{17}ClO_5$
**64**	(1′*S*)-1′-*O*-methyl-7-chloroaverantin	$C_{21}H_{21}ClO_7$	**173**	Pestalochromone C	$C_{11}H_{11}ClO_3$
**65**	(1′*S*)-6,1′-*O*,*O*-dimethyl-7-chloroaverantin	$C_{22}H_{23}ClO_7$	**174**	Pestalotiopene C	$C_{27}H_{33}ClO_{10}$
**66**	(1′*S*)-7-chloroaverantin-1′-butyl ether	$C_{24}H_{27}ClO_7$	**175**	Paradictyoarthrin A	$C_{16}H_{19}ClO_8$
**67**	7-chloroaverythrin	$C_{20}H_{17}ClO_6$	**176**	Chloropreussomerin A	$C_{21}H_{15}ClO_8$
**68**	6-*O*-methyl-7-chloroaverythrin	$C_{21}H_{19}ClO_6$	**177**	Chloropreussomerin B	$C_{22}H_{17}ClO_8$
**69**	(1′*S*)-6,1′-*O*,*O*-	$C_{22}H_{23}BrO_7$	**178**	Rhizovarin A	$C_{36}H_{42}ClNO_8$

	dimethyl-7-bromoaverantin				
**70**	(1′*S*)-6-*O*-methyl-7-bromoaverantin	$C_{21}H_{21}BrO_7$	**179**	Rhizovarin B	$C_{37}H_{44}ClNO_8$
**71**	5-chlorosclerotiamide	$C_{26}H_{28}ClN_3O_5$	**180**	Rhinomilisin A	$C_{30}H_{41}ClO_{10}$
**72**	5′-Hydroxychlorflavonin	$C_{18}H_{15}ClO_8$	**181**	Rhinomilisin B	$C_{15}H_{23}ClO_6$
**73**	7-chlorofolipastatin	$C_{23}H_{23}ClO_5$	**182**	Rhinomilisin B	$C_{16}H_{25}ClO_6$
**74**	Chloriolin A	$C_{14}H_{21}ClO_3$	**183**	Rhinomilisin I	$C_{15}H_{21}ClO_5$
**75**	Chloriolin B	$C_{22}H_{33}ClO_7$	**184**	Polyporapyranone D	$C_{11}H_7ClO_3$
**76**	Chloriolin C	$C_{22}H_{33}ClO_6$	**185**	Chlorogentisylquinone	$C_7H_5ClO_3$
**77**	Trichodenone B	$C_7H_9ClO_3$	**186**	Spiromastixone B	$C_{19}H_{19}ClO_5$
**78**	Trichodenone C	$C_7H_9ClO_2$	**187**	Spiromastixone C	$C_{19}H_{19}ClO_5$
**79**	Gymnastatin A	$C_{23}H_{31}Cl_2NO_4$	**188**	Spiromastixone D	$C_{19}H_{18}Cl_2O_5$
**80**	Gymnastatin B	$C_{24}H_{35}Cl_2NO_5$	**189**	Spiromastixone E	$C_{19}H_{18}Cl_2O_5$
**81**	Gymnastatin C	$C_{24}H_{37}Cl_2NO_6$	**190**	Spiromastixone F	$C_{19}H_{17}Cl_3O_5$
**82**	Gymnastatin D	$C_{22}H_{32}ClNO_5$	**191**	Spiromastixone G	$C_{20}H_{19}Cl_3O_5$
**83**	Gymnastatin E	$C_{23}H_{34}ClNO_5$	**192**	Spiromastixone H	$C_{19}H_{17}Cl_3O_5$
**84**	Gymnastatin F	$C_{25}H_{39}Cl_2NO_5$	**193**	Spiromastixone I	$C_{19}H_{16}Cl_4O_5$
**85**	Gymnastatin G	$C_{24}H_{38}ClNO_6$	**194**	Spiromastixone J	$C_{20}H_{18}Cl_4O_5$
**86**	Gymnastatin I	$C_{23}H_{31}Br_2NO_4$	**195**	Spiromastixone K	$C_{20}H_{19}Cl_3O_5$
**87**	Gymnastatin J	$C_{24}H_{35}Br_2NO_5$	**196**	Spiromastixone L	$C_{20}H_{18}Cl_4O_5$
**88**	Gymnastatin K	$C_{24}H_{37}Br_2NO_6$	**197**	Spiromastixone M	$C_{19}H_{17}Cl_3O_4$
**89**	Gymnastatin Q	$C_{24}H_{35}Cl_2NO_5$	**198**	Spiromastixone N	$C_{19}H_{17}Cl_3O_5$
**90**	Gymnastatin R	$C_{22}H_{31}Cl_2NO_4$	**199**	Spiromastixone O	$C_{19}H_{16}Cl_4O_5$
**91**	Dankastatin A	$C_{24}H_{35}Cl_2NO_5$	**200**	Emerixanthone A	$C_{26}H_{29}ClO_5$
**92**	Dankastatin B	$C_{23}H_{33}Cl_2NO_4$	**201**	Cladosporol G	$C_{20}H_{17}ClO_6$
**93**	Dankastatin C	$C_{25}H_{39}Cl_2NO_6$	**202**	Pestalone B	$C_{21}H_{23}BrO_6$
**94**	Chlorohydroaspyrone A	$C_9H_{13}ClO_4$	**203**	Pestalone C	$C_{20}H_{21}BrO_6$
**95**	Chlorohydroaspyrone B	$C_9H_{13}ClO_4$	**204**	Pestalone D	$C_{21}H_{22}Br_2O_6$
**96**	Chlorocylindrocarpol	$C_{23}H_{33}ClO_5$	**205**	Pestalone E	$C_{20}H_{21}ClO_6$
**97**	Acremofuranone A	$C_{22}H_{27}ClO_5$	**206**	Pestalone F	$C_{21}H_{23}ClO_6$
**98**	Acremofuranone B	$C_{23}H_{27}ClO_4$	**207**	Pestalone G	$C_{21}H_{22}BrClO_6$
**99**	5-Chloroacremine A	$C_{12}H_{17}ClO_4$	**208**	Pestalone H	$C_{21}H_{22}BrClO_6$

**100**	5-Chloroacremine H	$C_{12}H_{17}ClO_5$	**209**	N-glutarylchaetoviridin A	$C_{31}H_{38}ClNO_9$
**101**	Acremine O	$C_{12}H_{15}ClO_4$	**210**	N-glutarylchaetoviridin B	$C_{28}H_{30}ClNO_8$
**102**	Helicusin E	$C_{25}H_{29}ClO_9$	**211**	N-glutarylchaetoviridin C	$C_{30}H_{34}ClNO_8$
**103**	Isochromophilone X	$C_{27}H_{32}ClNO_7$	**212**	Neomangicol A	$C_{25}H_{37}ClO_5$
**104**	Isochromophilone XI	$C_{25}H_{29}ClO_8$	**213**	Neomangicol B	$C_{25}H_{37}BrO_5$
**105**	Minioluteumide A	$C_{22}H_{32}ClNO_5$	**214**	Chaephilone C	$C_{23}H_{27}ClO_7$
**106**	Stachybogrisephenone B	$C_{16}H_{15}ClO_6$	**215**	Chaetoviridide A	$C_{30}H_{31}ClN_2O_7$
**107**	(3*R*,4*S*)-4-hydroxy-6-methoxy-7-chloromellein	$C_{11}H_{11}ClO_5$	**216**	Chaetoviridide B	$C_{27}H_{34}ClNO_7$
**108**	Spiroxin A	$C_{19}H_9ClO_8$	**217**	Chaetoviridide C	$C_{25}H_{30}ClNO_6$
**109**	Spiroxin E	$C_{20}H_{10}Cl_2O_8$			
